# Spatiotemporal trends of ischemic stroke burden attributable to PM2.5 from 1990 to 2021

**DOI:** 10.3389/fpubh.2025.1608086

**Published:** 2025-07-16

**Authors:** Yunyan Lu, Yu Mao, Weiguo Liu, Tian Lan, Gaochen Lan

**Affiliations:** ^1^Department of Cardiology, The First People’s Hospital of Xiaoshan District, Xiaoshan Affiliated Hospital of Wenzhou Medical University, Hangzhou, Zhejiang, China; ^2^Department of Oncology, The People's Hospital of Jiangshan, Quzhou, Zhejiang, China; ^3^Department of Breast Surgery, Hangzhou TCM Hospital Affiliated to Zhejiang Chinese Medical University, Hangzhou Hospital of Traditional Chinese Medicine, Hangzhou, Zhejiang, China; ^4^Department of Oncology, The Second Affiliated Hospital of Fujian Medical University, Quanzhou, Fujian, China

**Keywords:** ischemic stroke, air pollution, particulate matter, Global Burden of Disease, disability-adjusted life years

## Abstract

**Background:**

To evaluate the spatiotemporal variation in ischemic stroke attributed to particulate matter 2.5 (PM2.5) on global, regional, and national scales from 1990 to 2021 is essential for mitigating air pollution and controlling ischemic stroke.

**Methods:**

The death and disability-adjusted life years (DALYs) were extracted from the Global Burden and Disease Study (GBD) 2021. We utilized joinpoint regression and decomposition analysis to assess PM2.5 exposure and pinpoint high-risk areas.

**Results:**

In 2021, PM2.5 caused approximately 0.90 million mortality and 18.29 million DALYs due to ischemic stroke worldwide. The age-standardized rates (ASRs) of ischemic stroke linked to ambient PM2.5 slightly declined, while those associated with household PM2.5 significantly decreased over the past 32 years. The burden of ischemic stroke attributable to ambient and household PM2.5 exhibited considerable heterogeneity across 204 countries. Household PM2.5 significantly affected ischemic stroke burdens in low Socio-demographic indices (SDI) regions, whereas ambient PM2.5 had a greater impact in middle, high-middle, and high SDI regions. In the regions with an SDI below 0.7, including Southern Sub-Saharan Africa and East, South, and Southeast Asia, there was a positive correlation between SDI and ASRs linked with ambient PM2.5. Notably, in the 65–95 age group, the age-specific rates associated with ambient PM2.5 showed a substantial decline among females, while the rates for males remained relatively stable.

**Conclusion:**

Our results presented that PM2.5 significantly affects global ischemic stroke burden, particularly among the male population and in low SDI regions. It highlighted the urgency of integrating PM2.5 reduction strategies with ischemic stroke prevention programs.

## Introduction

Stroke is the second leading cause of death worldwide, which is categorized into two primary types: ischemic stroke and hemorrhagic stroke (1). Ischemic stroke, accounting for approximately 87% of all stroke cases, is a critical cerebrovascular disease characterized by the abrupt disruption of blood flow to the brain (2). It is typically caused by thrombosis, embolism, and systemic hypoperfusion. Ischemic stroke is influenced by modifiable risk factors like hypertension, diabetes, physical inactivity, and tobacco use, as well as non-modifiable risk factors such as age, gender, and genetics (3, 4). The substantial disease burden necessitates comprehensive strategies to optimize stroke risk factor management.

Air pollution is an important modifiable risk factors, with particulate matter (PM) being one of the most detrimental pollutants to global health (5, 6). Numerous studies have explored the relationship between PM2.5 exposure (particulate matter with an aerodynamic diameter of less than 2.5 μm) and a spectrum of adverse health outcomes, including respiratory and cardiovascular diseases, primarily through mechanisms such as systemic inflammation and oxidative stress (7, 8). Long-term exposure to PM2.5 is significantly linked to increased incidence of ischemic stroke, with a 0.0091% elevation in disease risk observed for each 1 μg/m^3^ augmentation in PM2.5 concentration (9, 10). Consequently, a thorough evaluation and analysis of ischemic stroke attributable to PM2.5 is imperative for formulating public health strategies to reduce the impact of air pollution on the incidence and burden of ischemic stroke.

Previous studies on the spatiotemporal trend of ischemic stroke linked to PM2.5 have been conducted based on the Global Burden of Disease Study (GBD) 2019 (11–13). The COVID-19 pandemic that erupted at the end of 2019 led to unprecedented challenges, including overwhelming healthcare facilities, profound economic disruptions, and extensive social transformations (14). Meanwhile, the COVID-19 lockdown resulted in a decrease in PM2.5 concentrations while concurrently postponing the disease diagnosis and treatment, potentially altering epidemiological dynamics of ischemic stroke associated with PM2.5 (15). Ischemic stroke also represented a potential complication in the COVID-19 survivors within 9 months post-infection (16). Hence, to assess the influence of the COVID-19 pandemic on disease burden, we evaluated the spatiotemporal trend of PM2.5-related ischemic stroke based on the latest GBD 2021 study.

## Methods

### Data sources and collection

Our analysis was based on the most recent data sourced from the GBD 2021,^1^ which offered a comprehensive and comparative assessment of 288 estimates related to death causes, along with 87 risk factors, across 21 regions and 204 countries and territories during 1990–2021 (17). Our study specifically concentrated on ischemic strokes occurring in individuals aged 25 years and older. The participants were categorized into consecutive five-year age brackets (20–24, 25–29, 30–34… 90–94, and 95 + years). We also extracted the socio-demographic index (SDI) from the GBD 2021 study, a composite measure made for each country according to education, income per capita, and fertility rate. To classify all countries and territories, the SDI values in 2021 were utilized to divide them into five regions: low, low-middle, middle, high-middle, and high SDI regions.

The latest data comprised information on deaths, Disability-adjusted life year (DALYs) and corresponding age-standardized rate (ASR) for ischemic stroke, categorized by country, region, year, and age groups. DALYs were derived by aggregating the years lived with disability (YLDs) and the years of life lost (YLLs). YLDs were computed by multiplying the prevalence by the respective standardized disability weights for each health state. Additionally, YLLs were estimated based on a reference maximum observed life expectancy. ASRs were widely utilized to enhance the accuracy of disease burden comparisons, as they considered the age variation across populations.

Particulate matter pollution encompassed both ambient PM2.5 and household PM2.5. The former aspect, known as ambient particulate matter pollution, referred to as the annual mean concentration of particles with a diameter less than 2.5 micrometers, weighted by population (18). The latter aspect, commonly described as household air pollution stemming from solid fuels like coal, wood, dung, and charcoal, was estimated using both the proportion model and the PM2.5 mapping model (19). The impact of PM2.5 exposure on ischemic stroke was assessed using the proportional population attributable fraction, while simultaneously considering the potential effects of geography, year sex, and age.

### Statistical analyses

To evaluate the temporal trends, we implemented two approaches, including the estimated annual percentage change (EAPC) and the average annual percentage change (AAPC). A linear regression model was applied to compute the EAPC, and two formulas were set as followed.


ln(ASRs)=α+βX+ε

EAPC=100×(exp(β)–1)

In these equation, X denoted the calendar year, *ε* represented the error term, *β* referred to the coefficient. We also employed joinpoint analysis to compute annual percentage change (APC) along with its associated 95% confidence interval (CI). The AAPC was measured as the weighted average of APCs to succinctly describe the trends over a predetermined fixed period. The equation employed to compute AAPC was set as followed.


$$\text{AAPC}=\{\exp(\frac{\sum w_{i}b_{i}}{\sum w_{i}})\}\times 100$$

where *b* symbolized the coefficient for the segment, *w_i_* signified the length of the segment, *i* referred to the specific *i*th segment.

Decomposition analysis, motivated by Das Gupta’s method, was applied to gain insights into the disparities in disease burden across different regions (20). This approach attributed DALYs difference to the combined influences of three factors, such as population size, population aging, and epidemiological changes. We carried out the pearson’s correlation analysis to evaluate the relationship between ASRs and SDI. All statistics and visualization were executed via the R software (version 4.2.2). The establishment of statistical significance was predicated on a *p* value less than 0.05.

## Results

### Global ischemic stroke burden caused by particulate matter pollution

**Figure 1:**
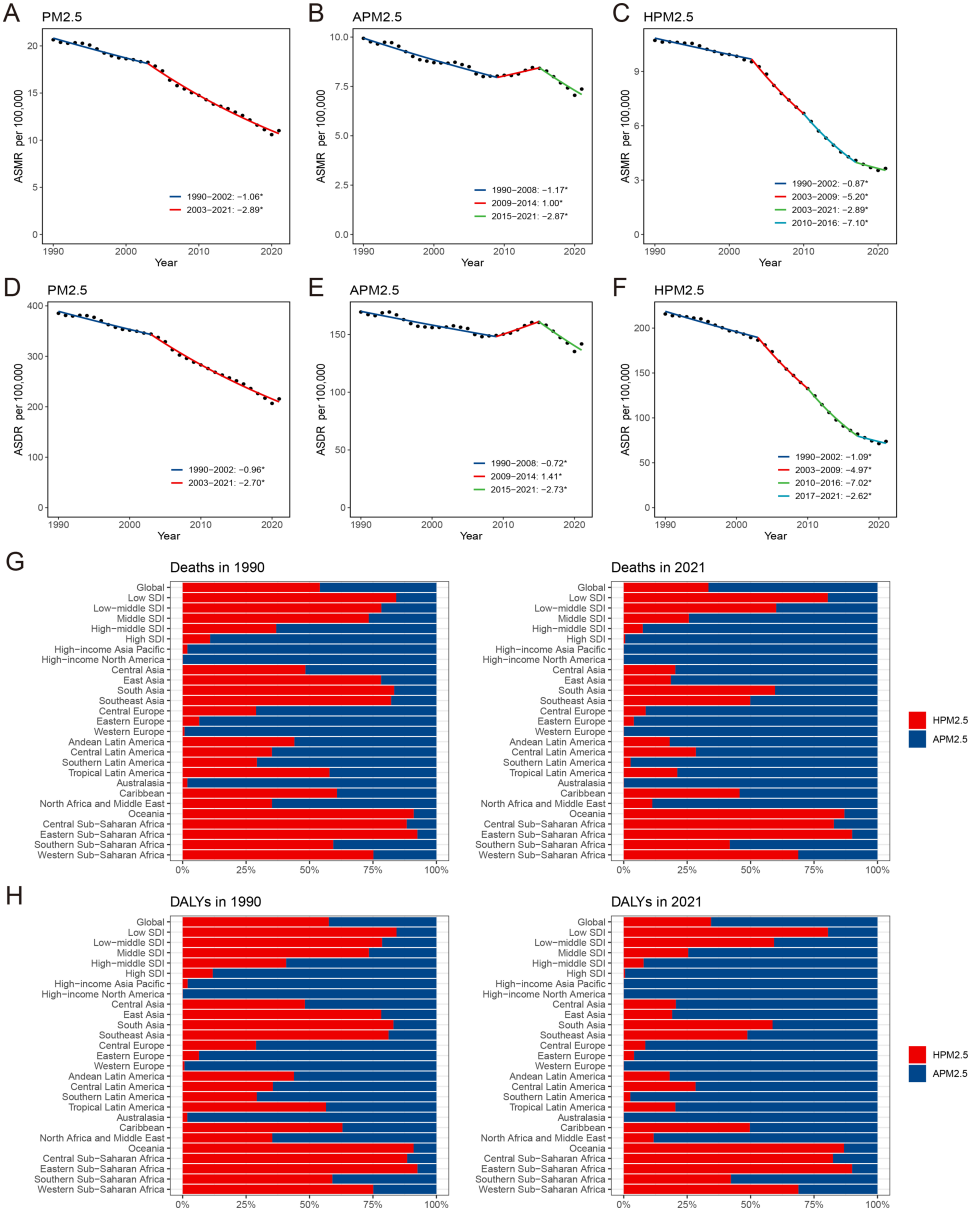
The global APCs in the age-standardized mortality and DALYs rate for ischemic stroke attributable to PM2.5 **(A,D)**, ambient PM2.5 **(B,E)** and household PM2.5 **(C,F)**, 1990–2021. Effect of ambient PM2.5 and household PM2.5 on the deaths **(G)** and DALYs **(H)** burden of PM2.5-related ischemic stroke. *p < 0.05, APC, annual percentage change; DALYs, disability-adjusted life-years; PM, particulate matter.

In 2021, the global impact of PM2.5 air pollution resulted in approximately 0.90 million ischemic stroke deaths and 18.29 million DALYs, representing an ASMR of 11.01 (95% UI 8.44–13.96) per 100,000 population and an ASDR of 215.64 (95% UI 168.84–266.03) per 100,000 population (Table 1). The ASRs in 2021 were less than those recorded in 1990, suggesting a downward trend as indicated by negative values for both EAPC (−2.19, 95%CI: −2.36 to −2.03 for ASMR; −2.04, 95%CI: −2.20 to −1.88 for ASDR) and AAPC (−2.13, 95%CI: −2.2 to −2.05 for ASMR; −1.98, 95%CI: −2.05 to −1.90 for ASDR) (Table 1; Supplementary Tables S1, S2; Supplementary Figures S1A,B). It indicated two periods of continuous decline in the ASMR and ASDR based on the joinpoint analysis (Figures 1A,D; Supplementary Tables S1, S2).

**Table 1:** The global burden of ischemic stroke attributable to PM2.5 air pollution in 1990 and 2021 and the temporal trends during 1990–2021.

Characteristics	1990				2021				EAPC of ASMR No. (95% CI)	EAPC of ASDR No. (95% CI)
	Death number No. × 10^4^ (95% UI)	DALY No. × 10^4^ (95% UI)	ASMR per 100,000 No. (95% UI)	ASDR per 100,000 No. (95% UI)	Death number No. × 10^4^ (95% UI)	DALY No. × 10^4^ (95% UI)	ASMR per 100,000 No. (95% UI)	ASDR per 100,000 No. (95% UI)		
Global	68.12 (53.71–83.67)	1414.14 (1128.47–1705.67)	20.65 (16.22–25.54)	385.24 (306.72–467.72)	90.56 (69.48–114.48)	1829.54 (1432.50–2254.14)	11.01 (8.44–13.96)	215.64 (168.84–266.03)	−2.19 (−2.36 to −2.03)	−2.04 (−2.20 to −1.88)
Female	36.83 (29.02–46.44)	720.70 (573.28–888.26)	19.10 (14.95–24.08)	351.11 (279.21–434.20)	43.80 (34.19–55.84)	850.90 (668.61–1070.05)	9.34 (7.30–11.91)	183.48 (144.22–230.80)	−2.52 (−2.67 to −2.36)	−2.31 (−2.45 to −2.16)
Male	31.29 (24.34–38.52)	693.44 (544.19–848.00)	22.68 (17.65–28.14)	427.43 (334.03–522.60)	46.76 (35.27–59.32)	978.63 (745.94–1222.81)	13.18 (9.97–16.74)	254.32 (193.91–317.98)	−1.86 (−2.05 to −1.67)	−1.79 (−1.97 to −1.61)
Low SDI	4.30 (3.32–5.62)	98.91 (77.33–127.89)	28.47 (22.04–36.75)	528.58 (412.86–669.59)	8.32 (6.54–10.67)	185.26 (146.06–234.85)	23.70 (18.67–30.13)	433.63 (345.01–543.78)	−0.60 (−0.67 to −0.53)	−0.70 (−0.75 to −0.64)
Low-middle SDI	11.55 (8.99–14.29)	255.06 (199.98–312.98)	26.37 (20.38–32.22)	487.32 (382.74–598.01)	22.27 (17.78–27.21)	465.88 (369.78–570.68)	19.43 (15.40–23.66)	358.32 (285.59–437.76)	−1.04 (−1.14 to −0.95)	−1.06 (−1.15 to −0.97)
Middle SDI	19.95 (15.85–24.62)	443.22 (356.04–541.88)	27.17 (21.51–33.58)	500.01 (401.24–612.41)	33.17 (24.44–43.58)	674.25 (497.43–871.68)	14.57 (10.74–19.19)	270.23 (199.58–349.64)	−2.06 (−2.32 to −1.80)	−2.03 (−2.27 to −1.80)
High-middle SDI	23.06 (16.75–30.03)	455.54 (333.77–581.56)	28.35 (20.42–37.17)	499.61 (364.15–642.44)	21.51 (15.99–28.30)	406.48 (302.51–531.52)	11.10 (8.24–14.60)	206.36 (153.81–269.72)	−3.39 (−3.68 to −3.10)	−3.21 (−3.49 to −2.93)
High SDI	9.16 (6.13–12.99)	159.50 (111.58–223.12)	8.21 (5.47–11.67)	141.77 (98.81–198.11)	5.22 (3.78–6.88)	96.30 (72.21–124.09)	2.08 (1.53–2.74)	43.95 (33.08–56.15)	−4.67 (−4.79 to −4.56)	−4.00 (−4.10 to −3.90)
High-income Asia Pacific	1.17 (0.33–2.38)	22.13 (6.37–43.19)	6.84 (1.86–14.0)	118.36 (33.63–232.34)	1.21 (0.70–1.83)	20.60 (12.26–30.76)	1.75 (1.03–2.6)	37.98 (23.31–56.43)	−4.89 (−5.27 to −4.51)	−4.14 (−4.52 to −3.75)
High-income North America	1.03 (0.40–1.84)	18.66 (7.20–32.58)	2.77 (1.06–4.92)	50.81 (19.59–88.76)	0.40 (0.20–0.67)	7.51 (3.60–12.32)	0.53 (0.26–0.89)	10.95 (5.24–18.00)	−6.08 (−6.54 to −5.62)	−5.66 (−6.09 to −5.23)
Central Asia	0.82 (0.46–1.21)	17.65 (10.10–26.05)	20.23 (11.39–30.17)	400.79 (229.00–591.62)	0.96 (0.71–1.25)	21.27 (15.76–27.28)	15.02 (11.05–19.63)	290.60 (214.85–373.60)	−1.63 (−1.98 to −1.27)	−1.71 (−2.07 to −1.34)
East Asia	20.95 (16.37–26.46)	478.39 (380.38–594.32)	35.24 (27.65–43.96)	648.02 (510.41–804.86)	36.68 (26.58–48.37)	732.01 (535.30–957.62)	19.21 (13.98–25.30)	352.95 (257.75–460.69)	−1.92 (−2.32 to −1.51)	−1.92 (−2.26 to −1.57)
South Asia	8.40 (6.35–11.01)	189.00 (144.45–247.13)	21.20 (16.08–27.44)	391.60 (298.90–508.10)	18.43 (14.41–23.90)	379.45 (298.72–499.79)	15.84 (12.45–20.15)	285.73 (225.61–369.84)	−1.08 (−1.20 to −0.95)	−1.16 (−1.26 to −1.07)
Southeast Asia	5.92 (4.58–7.19)	130.23 (103.24–155.20)	32.89 (25.52–39.91)	603.05 (477.48–717.64)	8.94 (6.03–12.29)	187.69 (129.04–257.81)	17.92 (12.17–24.51)	326.71 (224.42–447.81)	−2.00 (−2.33 to −1.67)	−2.05 (−2.34 to −1.75)
Central Europe	4.68 (2.75–6.56)	82.24 (48.31–116.57)	36.48 (21.47–51.21)	591.05 (348.08–836.17)	2.29 (1.69–3.27)	36.83 (27.14–53.24)	9.36 (6.91–13.38)	155.80 (114.61–225.77)	−4.73 (−5.04 to −4.42)	−4.66 (−4.97 to −4.35)
Eastern Europe	8.41 (4.20–13.05)	153.35 (77.64–238.49)	34.91 (17.31–54.21)	583.72 (295.95–908.41)	2.90 (1.82–4.40)	50.41 (31.50–75.99)	8.03 (5.02–12.17)	140.97 (88.08–212.43)	−5.81 (−6.35 to −5.25)	−5.63 (−6.17 to −5.09)
Western Europe	5.67 (2.73–9.39)	86.78 (42.06–145.57)	9.34 (4.49–15.49)	142.17 (68.89–238.44)	1.49 (0.99–2.12)	22.38 (15.15–31.22)	1.15 (0.77–1.64)	19.86 (13.37–27.67)	−6.91 (−7.07 to −6.75)	−6.50 (−6.63 to −6.36)
Andean Latin America	0.23 (0.18–0.29)	4.49 (3.41–5.63)	13.55 (10.25–17.12)	238.81 (181.26–299.33)	0.20 (0.13–0.30)	3.77 (2.45–5.42)	3.71 (2.41–5.38)	66.09 (42.88–95.13)	−4.73 (−5.03 to −4.43)	−4.69 (−4.96 to −4.42)
Central Latin America	0.66 (0.43–0.91)	12.83 (8.41–17.88)	10.27 (6.63–14.07)	174.28 (113.97–242.26)	0.61 (0.41–0.88)	11.24 (7.62–15.98)	2.65 (1.79–3.83)	47.05 (31.90–66.95)	−4.52 (−4.67 to −4.36)	−4.43 (−4.57 to −4.28)
Southern Latin America	0.42 (0.23–0.64)	7.56 (4.02–11.60)	10.27 (5.53–15.67)	173.31 (92.16–265.98)	0.23 (0.14–0.36)	4.18 (2.52–6.38)	2.52 (1.54–3.82)	46.15 (27.83–70.52)	−4.32 (−4.57 to −4.06)	−4.19 (−4.44 to −3.94)
Tropical Latin America	1.21 (0.72–1.82)	23.41 (13.90–35.55)	17.94 (10.72–26.60)	298.24 (179.11–451.00)	0.73 (0.42–1.12)	13.05 (7.53–20.14)	3.02 (1.76–4.66)	52.49 (30.31–81.04)	−5.64 (−5.82 to −5.45)	−5.62 (−5.80 to −5.44)
Australasia	0.04 (0.00–0.11)	0.62 (0.02–1.80)	1.76 (0.06–5.18)	27.15 (0.92–78.97)	0.05 (0.03–0.07)	0.74 (0.43–1.12)	0.70 (0.40–1.07)	12.35 (7.10–18.65)	−3.62 (−4.14 to −3.11)	−3.23 (−3.80 to −2.66)
Caribbean	0.31 (0.21–0.44)	5.82 (4.03–8.21)	13.38 (9.12–19.26)	234.39 (161.76–331.84)	0.43 (0.29–0.60)	8.02 (5.41–11.10)	7.99 (5.42–11.04)	148.84 (100.51–206.04)	−1.46 (−1.59 to −1.33)	−1.30 (−1.44 to −1.17)
North Africa and Middle East	3.81 (2.90–4.79)	81.98 (62.81–102.90)	30.99 (23.59–38.83)	557.79 (426.67–699.27)	6.83 (5.14–8.36)	147.18 (111.08–180.69)	19.45 (14.79–24.12)	357.47 (270.62–434.47)	−1.45 (−1.53 to −1.37)	−1.40 (−1.49 to −1.31)
Oceania	0.03 (0.02–0.05)	0.89 (0.65–1.17)	20.81 (14.95–27.50)	393.88 (291.97–514.70)	0.07 (0.05–0.10)	1.80 (1.29–2.44)	16.24 (11.50–22.33)	305.69 (220.23–415.99)	−0.88 (−0.97 to −0.79)	−0.90 (−0.97 to −0.83)
Central Sub-Saharan Africa	0.41 (0.30–0.54)	9.93 (7.47–12.99)	31.31 (23.00–40.91)	582.11 (439.00–750.48)	0.81 (0.55–1.18)	18.63 (13.15–25.73)	25.92 (17.42–37.82)	462.79 (327.71–648.45)	−0.79 (−0.85 to −0.72)	−0.92 (−0.98 to −0.85)
Eastern Sub-Saharan Africa	1.24 (0.94–1.62)	29.15 (22.81–37.43)	25.91 (20.25–33.71)	485.63 (382.45–617.30)	2.52 (1.95–3.14)	57.63 (45.94–71.52)	22.61 (17.48–28.18)	422.04 (334.50–519.73)	−0.52 (−0.56 to −0.47)	−0.54 (−0.58 to −0.50)
Southern Sub-Saharan Africa	0.31 (0.22–0.41)	6.94 (5.06–8.95)	14.98 (10.60–19.67)	289.38 (210.02–374.85)	0.57 (0.41–0.75)	11.99 (8.81–15.78)	13.57 (9.77–18.02)	244.19 (178.23–320.76)	−0.21 (−0.78 to 0.36)	−0.48 (−0.97 to 0.02)
Western Sub-Saharan Africa	2.40 (1.77–3.19)	52.08 (39.54–68.98)	37.69 (27.64–49.78)	689.21 (525.51–901.26)	4.19 (3.20–5.35)	93.15 (71.87–117.92)	30.96 (23.68–39.26)	564.04 (438.70–713.78)	−0.63 (−0.74 to −0.52)	−0.65 (−0.77 to −0.54)

ASMR, age-standardized mortality rate; UI, uncertainty interval; DALYs, disability-adjusted life years; ASDR, age-standardized DALY rate; EAPC, estimated annual percentage change; CI, confidence interval; SDI, Socio-demographic Index.

The ASMR and ASDR of ambient PM2.5-related ischemic stroke exhibited a slight downward trend, as evidenced by the negative EAPC and AAPC (Supplementary Tables S3–S5). It indicated a similar change pattern for both ASMR and ASDR according to the joinpoint analysis (Figures 1B,E). Specifically, the ASR decreased from 1990 to 2008, increased from 2009 to 2014, and then declined again from 2015 to 2021. The ASRs of household PM2.5-associated ischemic stroke decreased staggeringly over past three decades (Figures 1C,F; Supplementary Table S6). In 2021, household PM2.5 contributed a notably lower proportion to the overall air pollution-related ischemic stroke burden compared to ambient PM2.5 (Figures 1G,H; Supplementary Figures S1C,D).

### Regional ischemic stroke burden caused by particulate matter pollution

**Figure 2:**
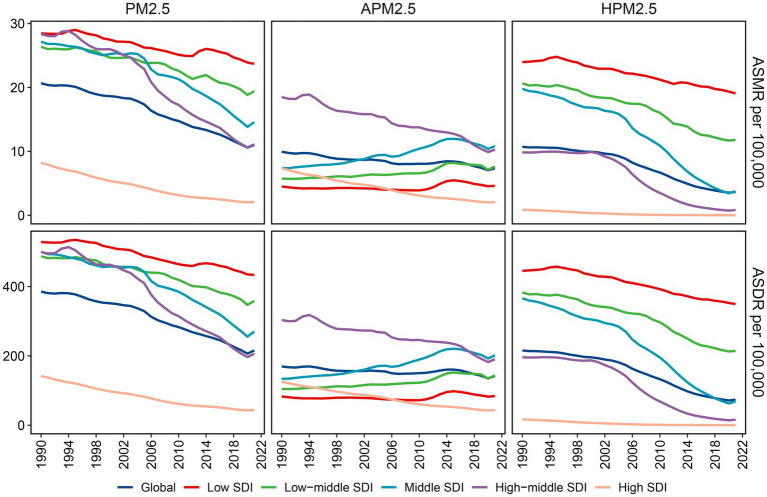
Variations in ischemic stroke ASMR and ASDR attributable to PM2.5, ambient PM2.5 and household PM2.5 across different SDI regions. ASMR: age-standardized mortality rate; ASDR: age-standardized DALYs rate; PM, particulate matter; SDI, socio-demographic index.

With respect to the SDI region, household PM2.5 had a more pronounced impact on the ischemic stroke burden in low SDI region, whereas ambient PM2.5 made a more dominant contribution in middle, high-middle, and high SDI regions (Figures 1G,H). In all five SDI regions, the proportion of ischemic stroke death and DALYs attributed to household PM2.5 showed a decrease in 2021 compared to those recorded in 1990, while ambient PM2.5-related percentage rose (Supplementary Figures S1C,D). For ischemic stroke attributed to PM2.5 or household PM2.5, the ASRs tended to be lower in regions with higher SDI (Table 1; Figure 2). Both ASMR and ASDR of household PM2.5-associated ischemic stroke approached zero in high SDI region (Supplementary Table S6). In the low, low-middle, and middle SDI regions, the ASRs of ambient PM2.5-related ischemic stroke increased slowly over time, supported by positive values for both AAPC and EAPC (Figure 2; Supplementary Tables S3–S5).

With respect to the GBD region, Africa suffered the heaviest ischemic stroke burden attributable to PM2.5 in 2021 (Table 1). While the highest ASMR and ASDR were manifested in the Africa, East Asia, and North Africa and Middle East. The distribution of ischemic stroke burden attributable to ambient PM2.5 and household PM2.5 varied among the GBD regions (Supplementary Figures S1C,D). Ambient PM2.5-related ischemic stroke exceeded 95% of the total burden in some high-income regions, namely High-income Asia Pacific, High-income North America, Western Europe, and Australasia (Figures 1G,H). A substantial proportion of the disease burden was attributed to household PM2.5 in Central, Eastern, and Western Sub-Saharan Africa, as well as Oceania. The contribution of household PM2.5 to both ischemic stroke mortality and DALYs declined across all GBD regions from 1990 to 2021.

In addition, there was a notable decline in both ASMR and ASDR for ischemic stroke associated with PM2.5 and household PM2.5 in almost all GBD regions (Table 1; Supplementary Tables S1–S4, S6). However, both ASMR and ASDR for ambient PM2.5-related ischemic stroke significantly increased in Oceania, Sub-Saharan Africa, Central Asia, East Asia, South Asia, and Southeast Asia (Supplementary Tables S3–S5).

### National ischemic stroke burden caused by particulate matter pollution

**Figure 3:**
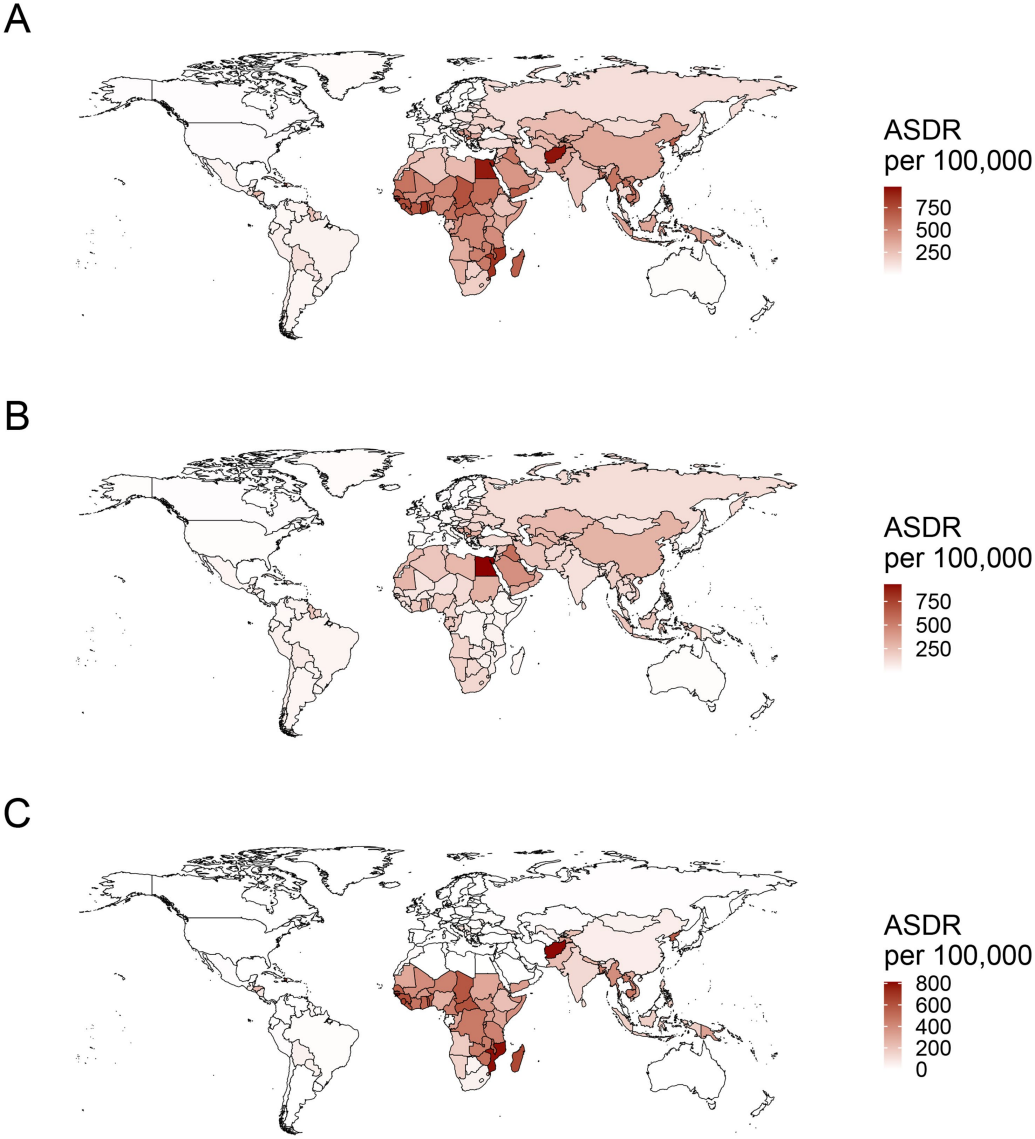
The worldwide distribution of ASDR for ischemic stroke attributed to PM2.5 **(A)**, ambient PM2.5 **(B)**, and household PM2.5 **(C)** air pollution in 2021. ASDR, age-standardized DALYs rate; PM, particulate matter.

**Figure 4:**
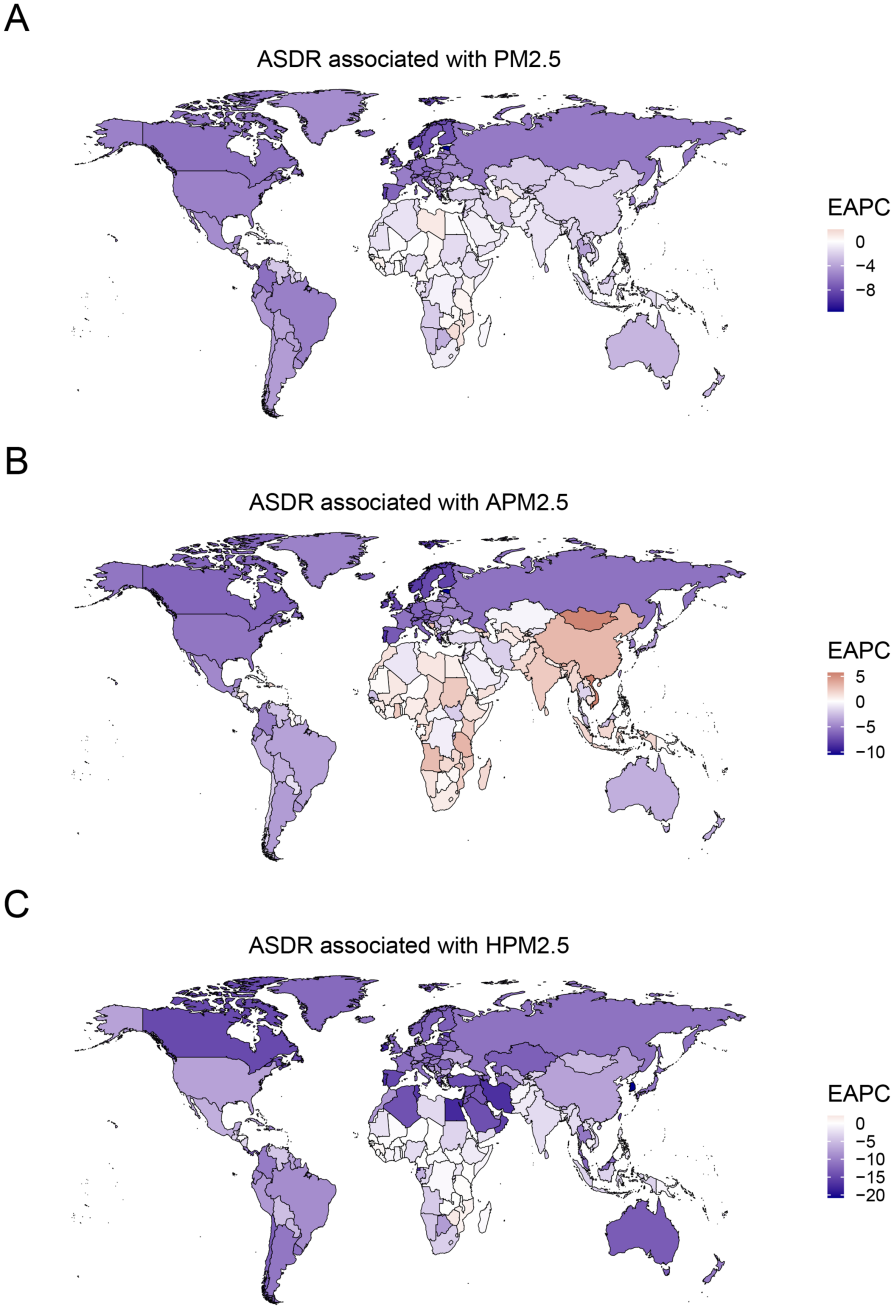
Temporal trends of ASDR for ischemic stroke attributed to PM2.5 **(A)**, ambient PM2.5 **(B)**, and household PM2.5 **(C)** air pollution during 1990–2021. ASDR, age-standardized DALYs rate; PM, particulate matter.

In the context of national level, the burden of PM2.5-related ischemic stroke displayed heterogeneity across 204 countries. ASMR and ASDR showed an upward trend in 15 and 12 countries, respectively. A downward trend was recorded for ASMR in 177 countries, and for ASDR in 179 countries (Supplementary Table S7). India, China, Bangladesh, and Indonesia ranked as the top four countries with the highest PM2.5-related ischemic stroke burden. The highest ASMR and ASDR were observed in several countries, such as Guinea-Bissau, Ghana, Gambia, Sierra Leone, Togo, Senegal, Benin, Liberia (West Africa), Egypt, Yemen (North Africa and Middle East), Haiti and Afghanistan (Figure 3A; Supplementary Figure S2A). Estonia and Portugal had the most minimal EAPC value, all less than −9.0 (Figure 4A; Supplementary Figures S3A, S4A, S5A). In contrast, the highest EAPC for both ASMR and ASDR were observed in Lesotho, Zimbabwe, Libya and Mozambique.

Ambient PM2.5-related ASMR decreased in 114 countries, increased in 69, and remained stable in 21 (Supplementary Table S8). ASDR declined in 116 countries, rose in 64, and stayed unchanged in 24. China, India, and Egypt were the top three countries with the highest burden of ambient PM2.5-related ischemic stroke. The maximal ASRs of ambient PM2.5-associated ischemic stroke were observed in the countries in North Africa and the Middle East, including Egypt, Iraq, Saudi Arabia, United Arab Emirates, Oman and Bahrain, along with those in Eastern Europe such as Bulgaria, North Macedonia, and Bosnia and Herzegovina (Figure 3B; Supplementary Figure S2B). Eastern and Southern Asia (Viet Nam, Mongolia, Bhutan, Timor-Leste, China, and India) and Africa (Cabo Verde, Tanzania, Lesotho, Angola, Sudan, Ghana, Kenya) exhibited the highest EAPC for both ASMR and ASDR (Figure 4B; Supplementary Figures S3B, S4B, S5B). More specifically, Viet Nam, Mongolia, and Equatorial Guinea emerged as the top three countries with the highest EAPC values above 4.0.

Over past 32 years, a declining trajectory was identified in household PM2.5-related ASMR across 184 countries and in household PM2.5-related ASDR across 185 countries (Supplementary Table S9). India, China, and Bangladesh were recognized as the top three countries bearing the highest cases of household PM2.5-related ischemic stroke. The majority of the top ten countries with the high ASRs consisted of Africa nations, such as Guinea-Bissau, Mozambique, Gambia, Sierra Leone, Madagascar, Togo, and Guinea (Figure 3C; Supplementary Figure S2C). Among the top ten countries with the lowest EAPC in ASRs, the majority were situated in Europe, encompassing Estonia, Portugal, Norway, Luxembourg, Austria, Finland, Sweden, United Kingdom, and Ireland (Figure 4C; Supplementary Figures S3C, S4C, S5C). The countries of Viet Nam, Mongolia, and Equatorial Guinea stood out as the top three with the highest EAPC in both ASMR and ASDR.

### Global ischemic stroke burden attributable to particulate matter pollution by age, gender, and SDI

**Figure 5:**
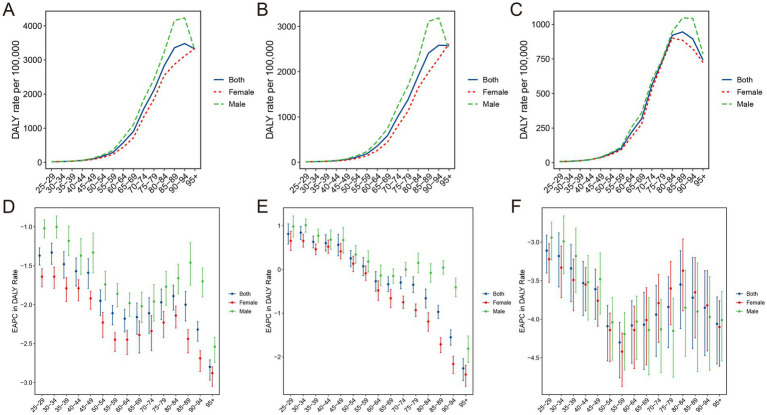
Age-specific DALYs rate of ischemic stroke attributed to PM2.5 **(A)**, ambient PM2.5 **(B)**, and household PM2.5 **(C)** air pollution and the corresponding changes in EAPC **(D–F)**, by sex, in 2021. DALYs, disability-adjusted life-years; PM, particulate matter; EAPC, estimated annual percentage change.

The age-specific death and DALYs rate for ischemic stroke associated with PM2.5 or ambient PM2.5 escalated with age increase (Figures 5A,B; Supplementary Figures S6A,B). For ischemic stroke associated with household PM2.5 exposure, the age-specific DALYs rate peaked in the 80–84 age group, while the age-specific mortality rate reached its highest point in the 90–94 age group (Figure 5C; Supplementary Figures S6C). Furthermore, the EAPCs of both death and DALYs rates exhibited negative values across all age groups for ischemic stroke attributable to PM2.5 or household PM2.5, signifying a notable declining trend (Figures 5D,F; Supplementary Figures S6D,F). For ambient PM2.5-associated ischemic stroke, the age-specific death and DALYs rate exhibited an upward trend among individual aged below 50, while demonstrating a decline among those aged above 60 (Figure 5E; Supplementary Figures S6E).

The findings of joinpoint analysis for ASMR and ASDR, stratified by gender, were shown in Supplementary Figure S7. Contrary to the dramatic drop in ASRs for ischemic stroke attributed to PM2.5 or household PM2.5, the ASRs trend for ambient PM2.5-related ischemic stroke remained relatively constant in the male populations. Additionally, the second segment of the ASRs temporal trends increased significantly in ischemic stroke associated with ambient PM2.5 for both female and male populations. For both ambient and household PM2.5, the mortality and DALYs rates were significantly higher in males than in females (Figures 5B,C; Supplementary Figures S6B,C). Interestingly, among individuals aged below 80, the mortality and DALYs rates for ischemic stroke attributed to household PM2.5 were nearly identical between males and females (Figure 5C; Supplementary Figure S6C). In the 65–95 age group, the age-specific rates of ambient PM2.5-related ischemic stroke exhibited a significant decline among females, whereas males demonstrated a relatively stable trend.

**Figure 6:**
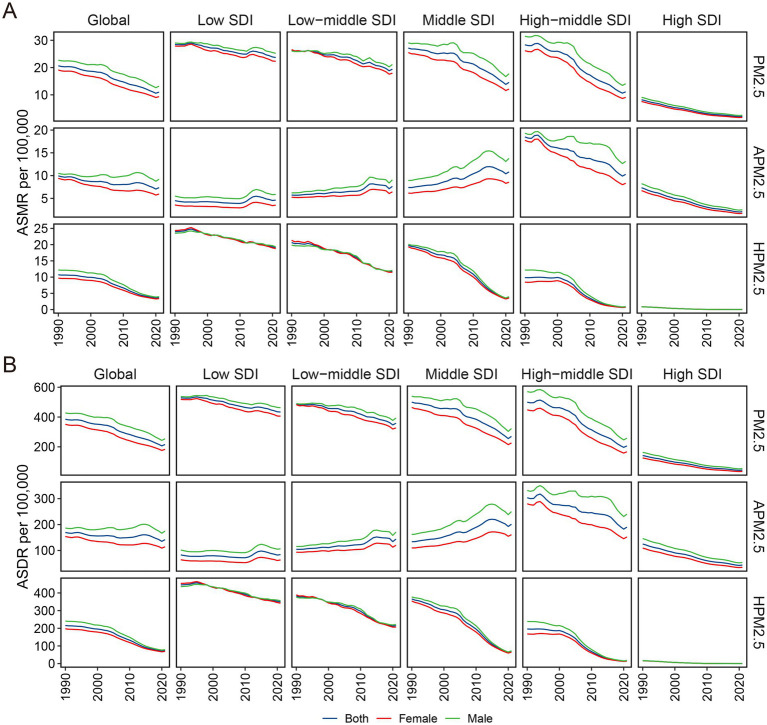
Sex disparity in ASMR **(A)** and ASDR **(B)** for ischemic stroke attributable to PM2.5, ambient PM2.5, and household PM2.5 air pollution across SDI regions. ASMR, age-standardized mortality rate; ASDR, age-standardized DALYs rate; PM, particulate matter; SDI, socio-demographic index.

For ischemic stroke associated with PM2.5 or household PM2.5, both ASMR and ASDR decreased with increased SDI (Figures 6A,B). These observations were consistent that in the high-middle and high SDI regions for ischemic stroke associated with ambient PM2.5. However, in the regions with low, low-middle, and middle SDI, the ASRs related to ambient PM2.5 demonstrated an upward trend corresponding to the SDI rise. In the GBD regions with an SDI below 0.7, including Southern Sub-Saharan Africa and East, South, and Southeast Asia, a positive correlation was identified between SDI and ASMR linked with ambient PM2.5 (Supplementary Figure S8). The pattern of ASDR based on SDI at the regional level was consistent with that of ASMR (Supplementary Figure S9).

There was an inverse relationship between ASRs and SDI across 204 nations for both ischemic stroke attributed to PM2.5 (*R* = −0.51, *p* < 0.01 for ASMR in 1990; *R* = −0.82, *p* < 0.01 for ASMR in 2021; *R* = −0.57, *p* < 0.01 for ASDR in 1990; *R* = −0.75, *p* < 0.01 for ASDR in 2021) and household PM2.5 (*R* = −0.75, *p* < 0.01 for ASMR in 1990; *R* = −0.82, *p* < 0.01 for ASMR in 2021; *R* = −0.77, *p* < 0.01 for ASDR in 1990; *R* = −0.82, *p* < 0.01 for ASDR in 2021) (Supplementary Figures S10A,C, S11A,C, S12A,C, S13A,C). Both in 1990 and 2021, a unimodal curve was perceptible when examining the correlation between SDI and ASRs for ischemic stroke associated with ambient PM2.5. It is noteworthy that over this period, the peak of the correlation curve underwent a transition, moving from 0.8 down to 0.7 (Supplementary Figures S10B, S11B, S12B, S13B).

### The decomposition of the change in ischemic stroke attributed to particulate matter pollution

**Figure 7:**
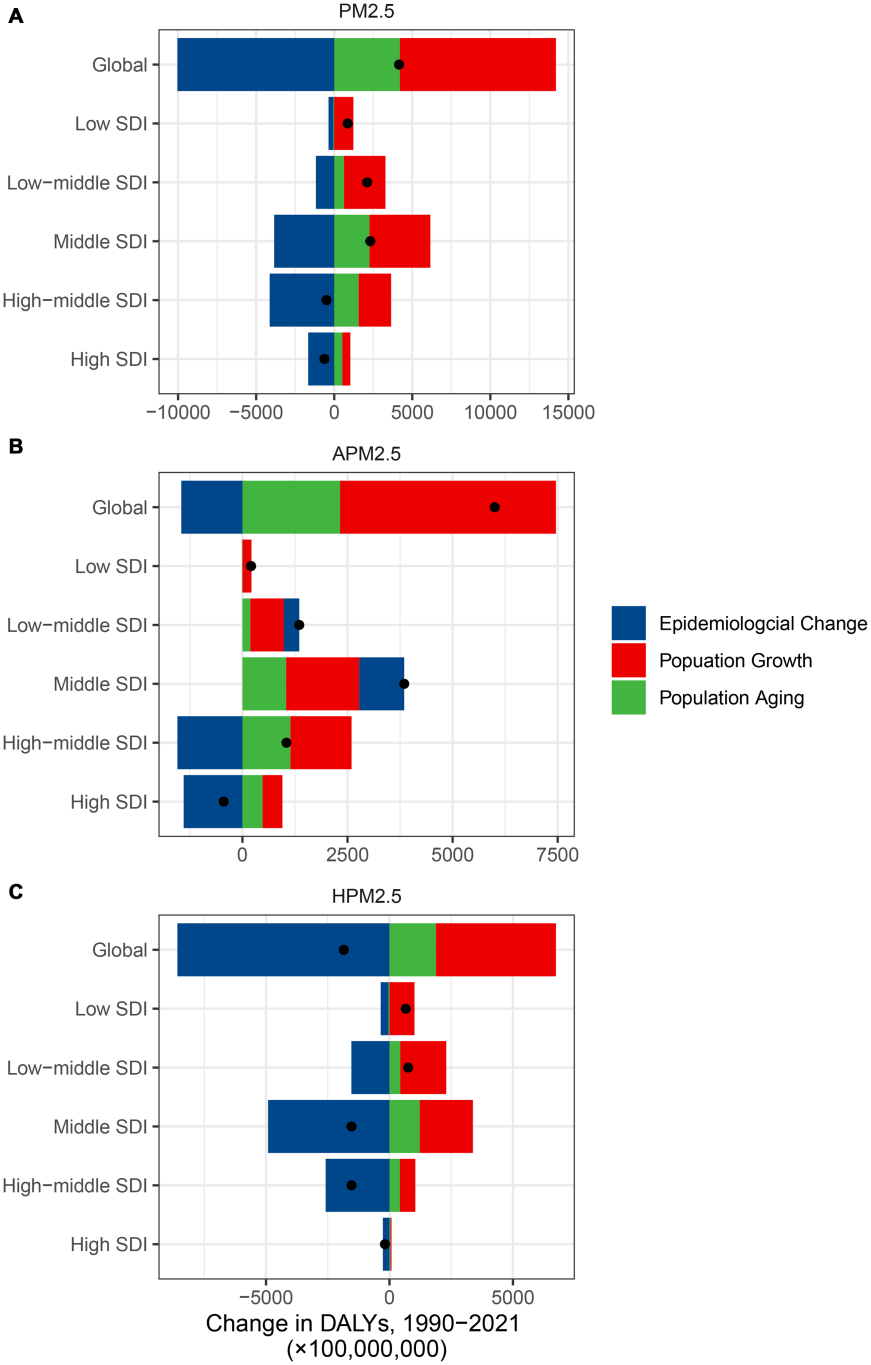
The contributions of three population-level factors to the DALYs change for ischemic stroke attributed to PM2.5 **(A)**, ambient PM2.5 **(B)**, and household PM2.5 **(C)** air pollution from 1990 to 2021 according to the decomposition analysis DALYs, disability-adjusted life years; PM, particulate matter pollution; SDI, sociodemographic index.

It presented a substantial worldwide escalation in DALYs attributed to PM2.5 and ambient PM2.5, but a significant reduction in DALYs linked to household PM2.5 (Figure 7; Supplementary Table S10). In the low and low-middle SDI regions, the DALYs increase of ischemic stroke related to PM2.5 and household PM2.5 was primarily driven by population growth and aging. The effect of epidemiological changes on DALYs growth showed a negative impact. All epidemiological changes, population growth and aging led to a considerable DALYs escalation linked to ambient PM2.5 across the low, low-middle, and middle SDI regions. In the high SDI region, the epidemiological changes led to a considerable DALYs reduction.

## Discussion

In the study, we conducted a comprehensive evaluation of the global, regional, and national burden and temporal trends of PM2.5-related ischemic stroke, stratifying the analysis by SDI, age, and sex. The findings highlighted the critical impact of particulate matter pollution on ischemic stroke, offering valuable scientific insights to guide policymakers in formulating effective preventive and remedial approaches.

In contrast to the dramatic decline in household PM2.5-related ASRs, it presented a more gradual decrease of ambient PM2.5-related ASRs over the past 32 years. According to the decomposition analysis, population growth and aging contributed to a measurable increase of DALYs associated with ambient PM2.5, whereas the epidemiological changes resulted in a considerable household PM2.5-associated DALYs reduction. Several underlying factors may elucidate this discrepancy. Firstly, the widespread adoption of cleaner fuels in household, such as ethanol, liquefied petroleum gas, electrification, and solar energy, could markedly reduce indoor particulate matter levels (21). Cookstove intervention programs, such as clean fuels, proper ventilation, and permeable construction material, may serve as another potential approach in reducing the disease burden associated with household PM2.5, especially in low SDI regions (22, 23). Secondly, the Environmental Kuznets Curve hypothesis suggested that environmental degradation escalated with industrialization and urbanization initially but declined as economies reach a certain income level, forming an inverted U-shape (24). The increase in population, road density and trip activity during urbanization had a significantly positive impact on PM2.5 concentrations (25). The emissions of sulfur dioxide, elemental carbon, and nitrates comprised the principal components of particulate matter, which proved to be detrimental (26). A reduction in these pollutants originating from mobile, point, area, and non-road sources could serve as a guiding principle for improving air quality management and reducing ischemic stroke mortality. During the COVID-19 pandemic, the enhancement of air quality was regarded as a benefit of the lockdown, resulting from the cessation of anthropogenic emissions (27). As the pandemic came to an end and society resumed its customary rhythm, there was a potential for a rise in air pollution. An economically sustainable “green recovery” plan should be more effectively implemented in the post-COVID-19 era, given the observed slight increase in the ASRs linked to both ambient and household PM2.5 during 2020–2021. The extensive adoption of telemedicine prompted by COVID-19 may be a crucial strategy for treating patients with PM2.5-related ischemic strokes and minimizing our carbon footprint (28).

SDI discrepancies led to varying disease burdens. There was a notable correlation between ASRs and SDI in the study. The ASRs associated with PM2.5 in the low SDI regions exceeded those in the high SDI regions. We also presented a modest upward trend of ambient PM2.5-related ASRs across the regions with low, low-middle, and middle SDI. It may be attributed to insufficient medical resource, limited health awareness, and low economic affordability in the low SDI regions (29, 30). In addition, economies in low SDI regions tended to depend more heavily on polluting industries and technologies, which may be one potential explanation (31). Low-income and middle-income countries allocated fewer resources to pollution control compared to high-income countries (32). Formulating tailored policies meticulously for various SDI regions to combat air pollution may be a more rational and influential strategy. Historical experiences from high-income countries could also help to inform potential evidence-based solutions, including medical specialization policy, green infrastructure, emissions-based air control measures, and zoning laws (33, 34). Nations across the world must collaborate closely, for air pollution in one country can yield far-reaching effects on its neighboring states (35).

Regarding the ASRs associated with ambient PM2.5, a stable trend was noted in males compared with a dramatic downward trend in females. The EAPCs of age-specific rates attributable to ambient PM2.5 in males were higher than those observed in females. The differences in time-activity patterns may be one explanation. It was reported that women spent the majority of their time at home, dedicating 83% of their daytime, in contrast to 57% for men (36). The sex-specific differences could also be attributed to variations in social functioning and biological distinctions between females and males (37). The biological factors related to sex, including hormonal profiles, lung capacity, bronchial hyperresponsiveness, and tobacco exposure, were distinct between males and females (38). However, the definitive cause of discrepancies by sex remained ambiguous, necessitating further investigation.

An approximate 10% increase in stroke mortality was observed with the rise in PM2.5 levels (39). There were some molecular biological mechanisms linking stroke and PM2.5. Diabetes, hypertension, hyperlipidemia, and atrial fibrillation, which were common risk factors for ischemic stroke, exhibited a significant association with PM2.5 exposure (10). The elevation of platelet count and DNA methylation induced by PM2.5 exposure increased the thrombosis risk through the activation of megakaryocytes, potentially representing one fundamental pathogenesis of ischemic stroke associated with PM2.5 (40). Additionally, the detrimental effects of PM2.5 on endothelial cells were mediated by inflammatory responses, oxidative stress, coagulation activation, autophagy, and ferroptosis (10). Some inflammatory factors, including IL-6 and IL-17, exacerbated leukocyte proliferation and inflammatory responses, thereby increasing the cerebral infarct area by activating the nuclear factor-kappa B and mitogen-activated protein kinase signaling pathways (41, 42). Inhaled particulate matter also can provoke inflammation in the lung, gut, and brain through the generation of reactive oxygen species production, neuroinflammation, microglial activation, and neuronal damage (43). Furthermore, the blood–brain barrier and the nasal mucosa-olfactory bulb pathway were two primary routes by which PM2.5 can infiltrate the brain (44, 45). PM2.5 can induce dysfunction in brain endothelial cells and exacerbate neurovascular injury through the mediation of perivascular macrophages (46). The definite mechanism in molecular gene level required further verification in future researches.

In this study, we conducted a thorough investigation into the spatiotemporal burden of ischemic strokes associated with PM2.5 based on the latest GBD 2021 study. However, there were several inevitable limitations. First, the composition of PM2.5 is intricate, encompassing diverse physical structures, chemical compositions, and sources of its constituents (47). The GBD 2021 study did not provide the detailed components of PM2.5, thus we did not incorporate the various constituents of PM2.5 into the analysis. Second, the considerable heterogeneity in data quality across regions may lead to uncertainty and bias of burden estimates, thereby impacting the generalizability of the findings. But two essential enhancements—the non-zero floor and the revised Bayesian algorithm—were implemented to alleviate stochastic variation in the GBD 2021 study (17). Third, several long-term cardiovascular and neurological complications were observed in survivors of COVID-19 (48, 49). The long-term impact of COVID-19 on ischemic stroke should be addressed in the forthcoming GBD 2023 study.

In conclusion, while the global burden of ischemic stroke attributable to both ambient and household PM2.5 declined from 1990 to 2021, there was still a notable increase of ischemic stroke associated with ambient PM2.5 in some countries, such as Viet Nam, Mongolia, and Equatorial Guinea. We explored the profound influence of ambient and household PM2.5 on ischemic stroke, reinforcing the urgent need of mitigating PM2.5 levels and enhancing public awareness. The results further furnished a robust epidemiological foundation for the prevention, control, and management of ambient PM2.5 pollution.

## Data Availability

Publicly available datasets were analyzed in this study. The data can be found here: https://ghdx.healthdata.org/gbd-results-tool. The R scripts of this study can be accessed on the website (https://github.com/lantian1986/ischemic_stroke_PM2.5).

## Footnotes

^1^ https://ghdx.healthdata.org/gbd-2021

## Supplementary material

SUPPLEMENTARY FIGURE S1 EAPC of ASMR **(A)** and ASDR **(B)** of PM2.5-associated ischemic stroke from 1990 to 2021. ASMR **(C)** and ASDR **(D)** of ischemic stroke associated with ambient PM2.5 and household PM2.5 in 1990 and 2021. EAPC, estimated annual percentage change; ASMR, age-standardized mortality rate; ASDR, age-standardized DALYs rate; PM, particulate matter.

SUPPLEMENTARY FIGURE S2 The worldwide distribution of ASMR for ischemic stroke attributed to PM2.5 **(A)**, ambient PM2.5 **(B)**, and household PM2.5 **(C)** air pollution in 2021. ASMR, age-standardized mortality rate; PM, particulate matter.

SUPPLEMENTARY FIGURE S3 The worldwide distribution of ASMR for ischemic stroke attributed to PM2.5 **(A)**, ambient PM2.5 **(B)**, and household PM2.5 **(C)** air pollution in 1990. ASMR, age-standardized mortality rate; PM, particulate matter.

SUPPLEMENTARY FIGURE S4 The worldwide distribution of ASDR for ischemic stroke attributed to PM2.5 **(A)**, ambient PM2.5 **(B)**, and household PM2.5 **(C)** air pollution in 1990. ASDR, age-standardized DALYs rate; PM, particulate matter.

SUPPLEMENTARY FIGURE S5 Temporal trends of ASMR for ischemic stroke attributed to PM2.5 **(A)**, ambient PM2.5 **(B)**, and household PM2.5 **(C)** air pollution during 1990-2021. ASMR, age-standardized mortality rate; PM, particulate matter.

SUPPLEMENTARY FIGURE S6 Age-specific death rate of ischemic stroke attributed to PM2.5 **(A)**, ambient PM2.5 **(B)**, and household PM2.5 **(C)** air pollution and the corresponding changes in EAPC **(D–F)**, by sex, in 2021. PM, particulate matter; EAPC, estimated annual percentage change.

SUPPLEMENTARY FIGURE S7 The global APCs of the ASMR and ASDR for ischemic stroke attributable to PM2.5, ambient PM2.5, and household PM2.5 air pollution, 1990-2021. Female ASMR **(A–C)** and ASDR **(D–F)**; male ASMR **(G–I)** and ASDR **(J–L)** **p*<0.05, APC: annual percentage change; ASMR, age-standardized mortality rate; ASDR, age-standardized DALYs rate; PM, particulate matter.

SUPPLEMENTARY FIGURE S8 The correlation between socio-demographic index and ASMR for ischemic stroke attributed to PM2.5, ambient PM2.5, and household PM2.5 air pollution across the GBD regions. ASMR, age-standardized mortality rate; PM, particulate matter; GBD, Global Burden of Disease.

SUPPLEMENTARY FIGURE S9 The correlation between socio-demographic index and ASDR for ischemic stroke attributed to PM2.5, ambient PM2.5, and household PM2.5 air pollution across the GBD regions. ASDR, age-standardized DALYs rate; PM, particulate matter; GBD, Global Burden of Disease.

SUPPLEMENTARY FIGURE S10 The relationship between socio-demographic index in 2021 and ASMR in 1990 for ischemic stroke attributed to PM2.5 **(A)**, ambient PM2.5 **(B)**, and household PM2.5 **(C)** air pollution across 204 countries or territories. ASMR, age-standardized mortality rate; PM, particulate matter.

SUPPLEMENTARY FIGURE S11 The relationship between socio-demographic index in 2021 and ASMR in 2021 for ischemic stroke attributed to PM2.5 **(A)**, ambient PM2.5 **(B)**, and household PM2.5 **(C)** air pollution across 204 countries or territories. ASMR, age-standardized mortality rate; PM, particulate matter.

SUPPLEMENTARY FIGURE S12 The relationship between socio-demographic index in 2021 and ASDR in 1990 for ischemic stroke attributed to PM2.5 **(A)**, ambient PM2.5 **(B)**, and household PM2.5 **(C)** air pollution across 204 countries or territories. ASDR, age-standardized DALYs rate; PM, particulate matter.

SUPPLEMENTARY FIGURE S13 The relationship between socio-demographic index in 2021 and ASDR in 2021 for ischemic stroke attributed to PM2.5 **(A)**, ambient PM2.5 **(B)**, and household PM2.5 **(C)** air pollution across 204 countries or territories. ASDR, age-standardized DALYs rate; PM, particulate matter.
