# Supplementary figures and images for: Spatiotemporal trends of ischemic stroke burden attributable to PM2.5 from 1990 to 2021
Source: Front Public Health. 2025 Jul 16;13:1608086. doi: 10.3389/fpubh.2025.1608086 (PMC12307462; doi:10.3389/fpubh.2025.1608086)

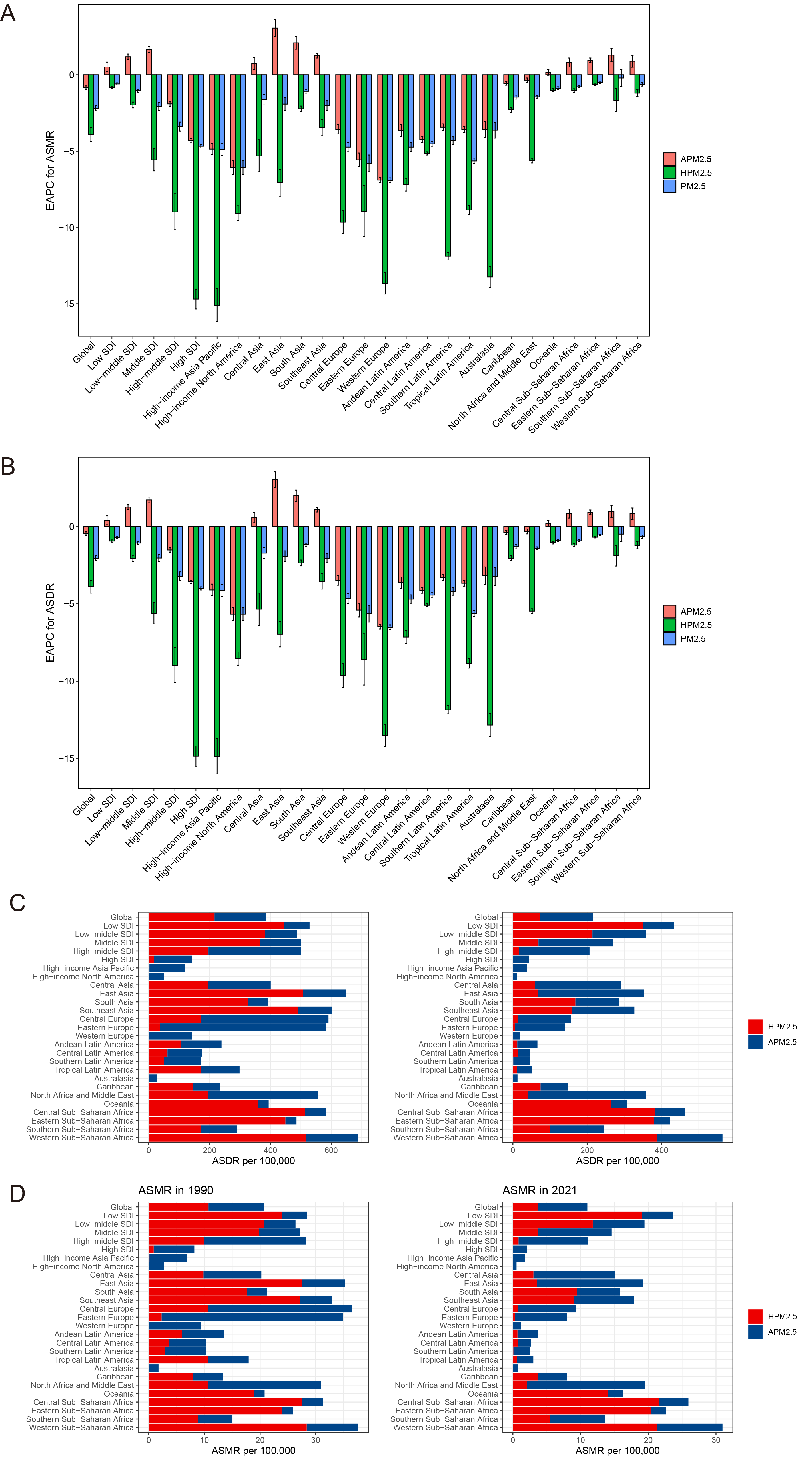

Supplement: SUPPLEMENTARY FIGURE S1 — EAPC of ASMR (A) and ASDR (B) of PM2.5-associated ischemic stroke from 1990 to 2021. ASMR (C) and ASDR (D) of ischemic stroke associated with ambient PM2.5 and household PM2.5 in 1990 and 2021. EAPC, estimated annual percentage change; ASMR, age-standardized mortality rate; ASDR, age-standardized DALYs rate; PM, particulate matter. [file Image_1.tif]

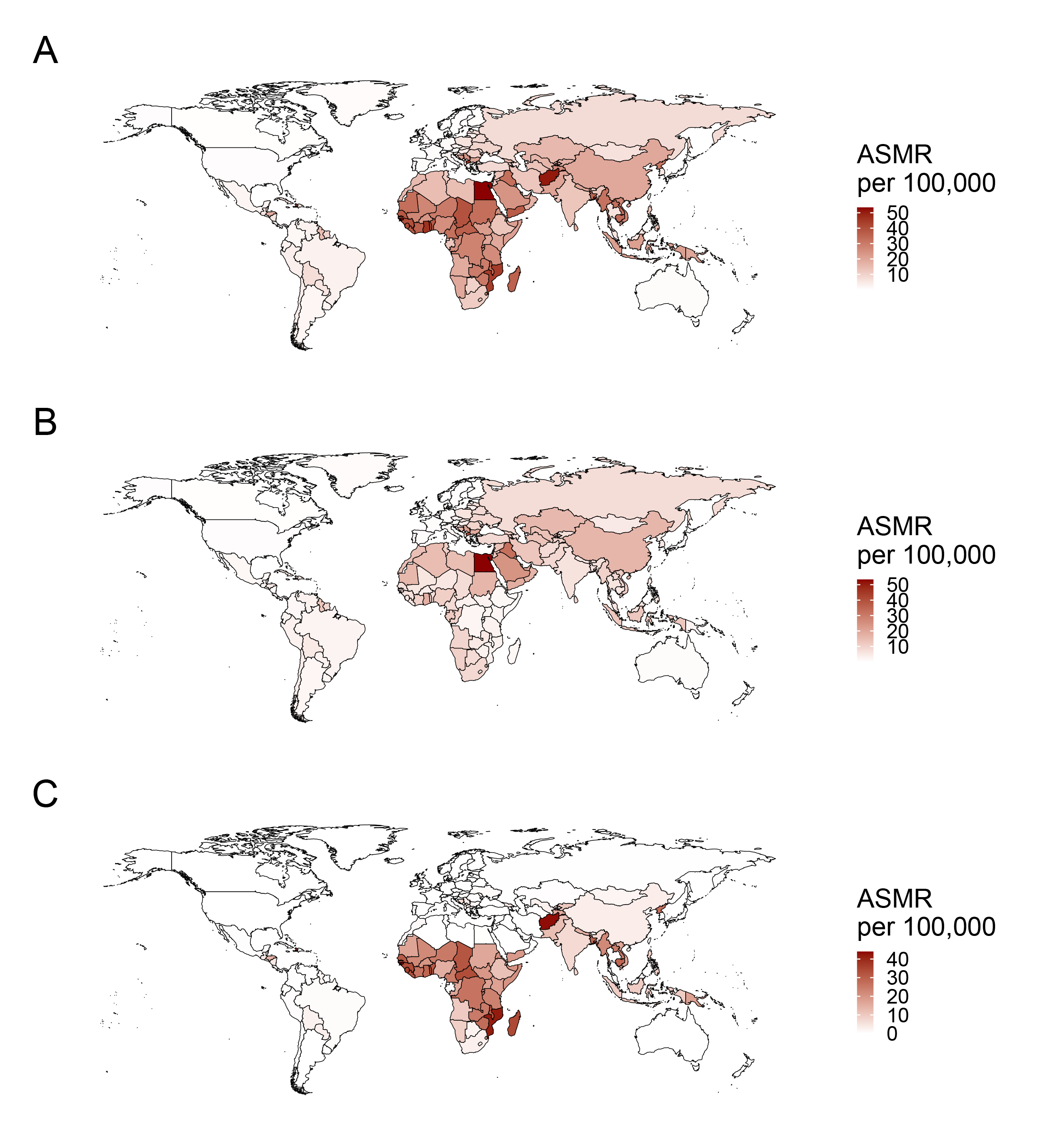

Supplement: SUPPLEMENTARY FIGURE S2 — The worldwide distribution of ASMR for ischemic stroke attributed to PM2.5 (A), ambient PM2.5 (B), and household PM2.5 (C) air pollution in 2021. ASMR, age-standardized mortality rate; PM, particulate matter. [file Image_2.tif]

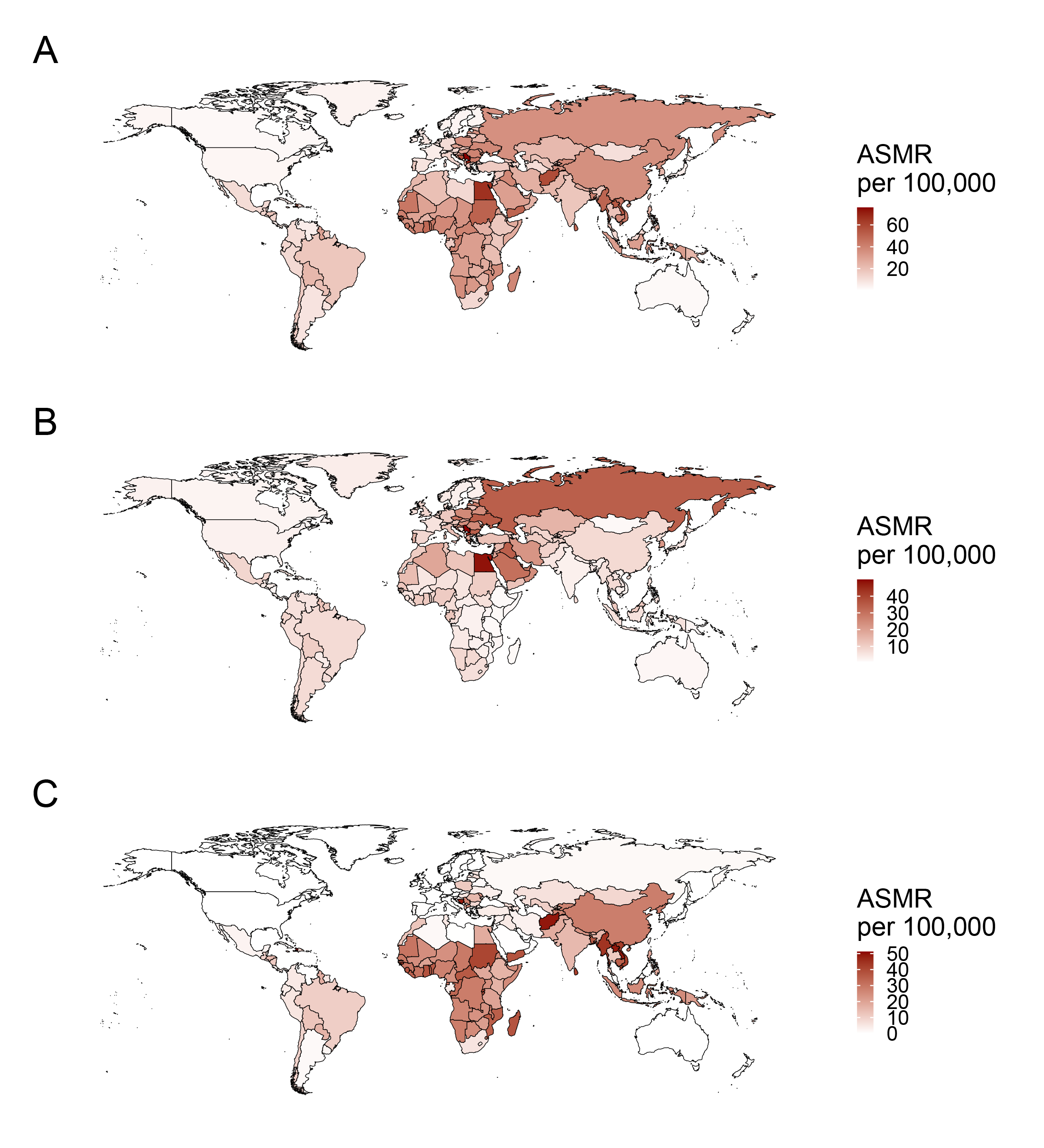

Supplement: SUPPLEMENTARY FIGURE S3 — The worldwide distribution of ASMR for ischemic stroke attributed to PM2.5 (A), ambient PM2.5 (B), and household PM2.5 (C) air pollution in 1990. ASMR, age-standardized mortality rate; PM, particulate matter. [file Image_3.tif]

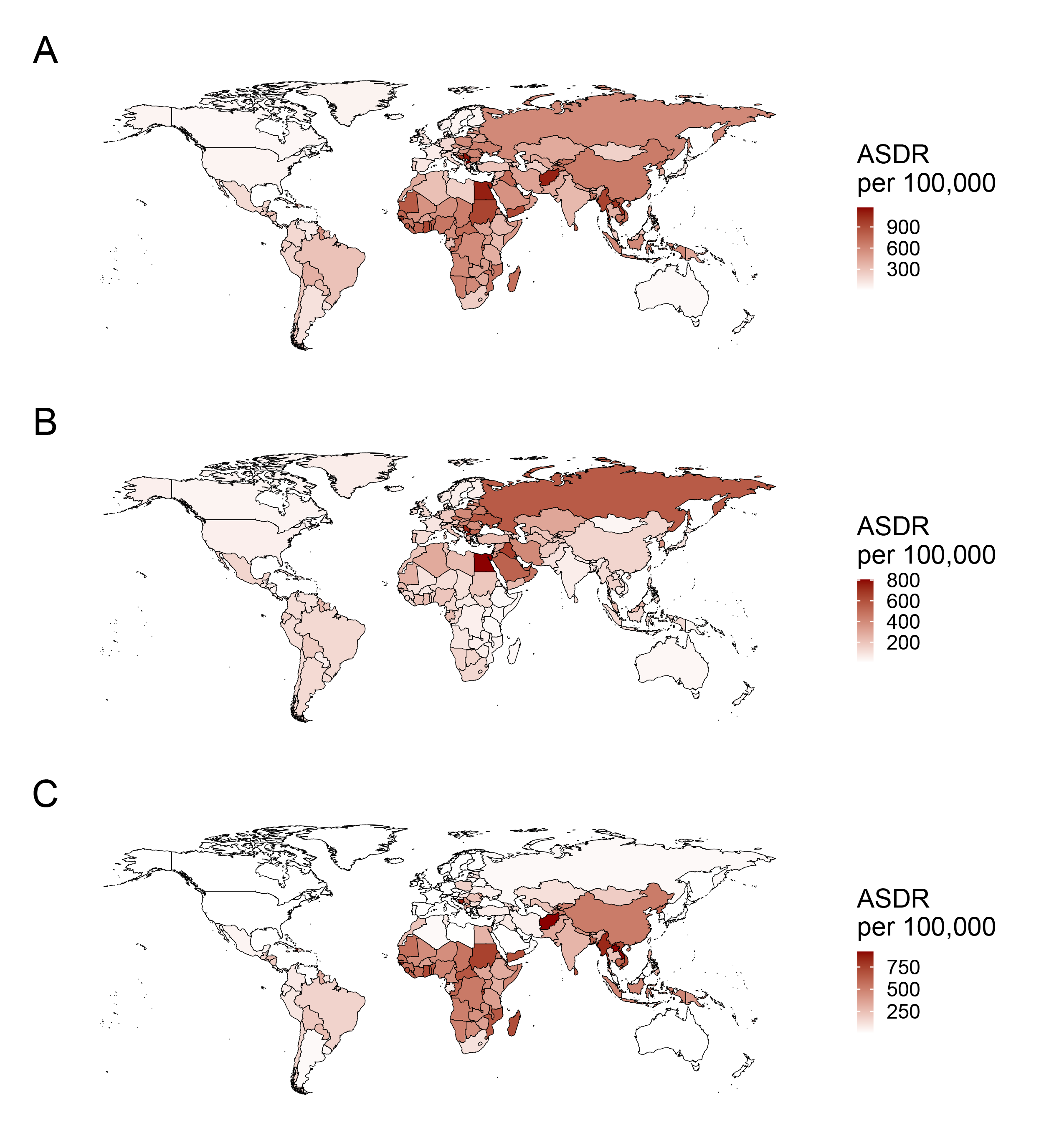

Supplement: SUPPLEMENTARY FIGURE S4 — The worldwide distribution of ASDR for ischemic stroke attributed to PM2.5 (A), ambient PM2.5 (B), and household PM2.5 (C) air pollution in 1990. ASDR, age-standardized DALYs rate; PM, particulate matter. [file Image_4.tif]

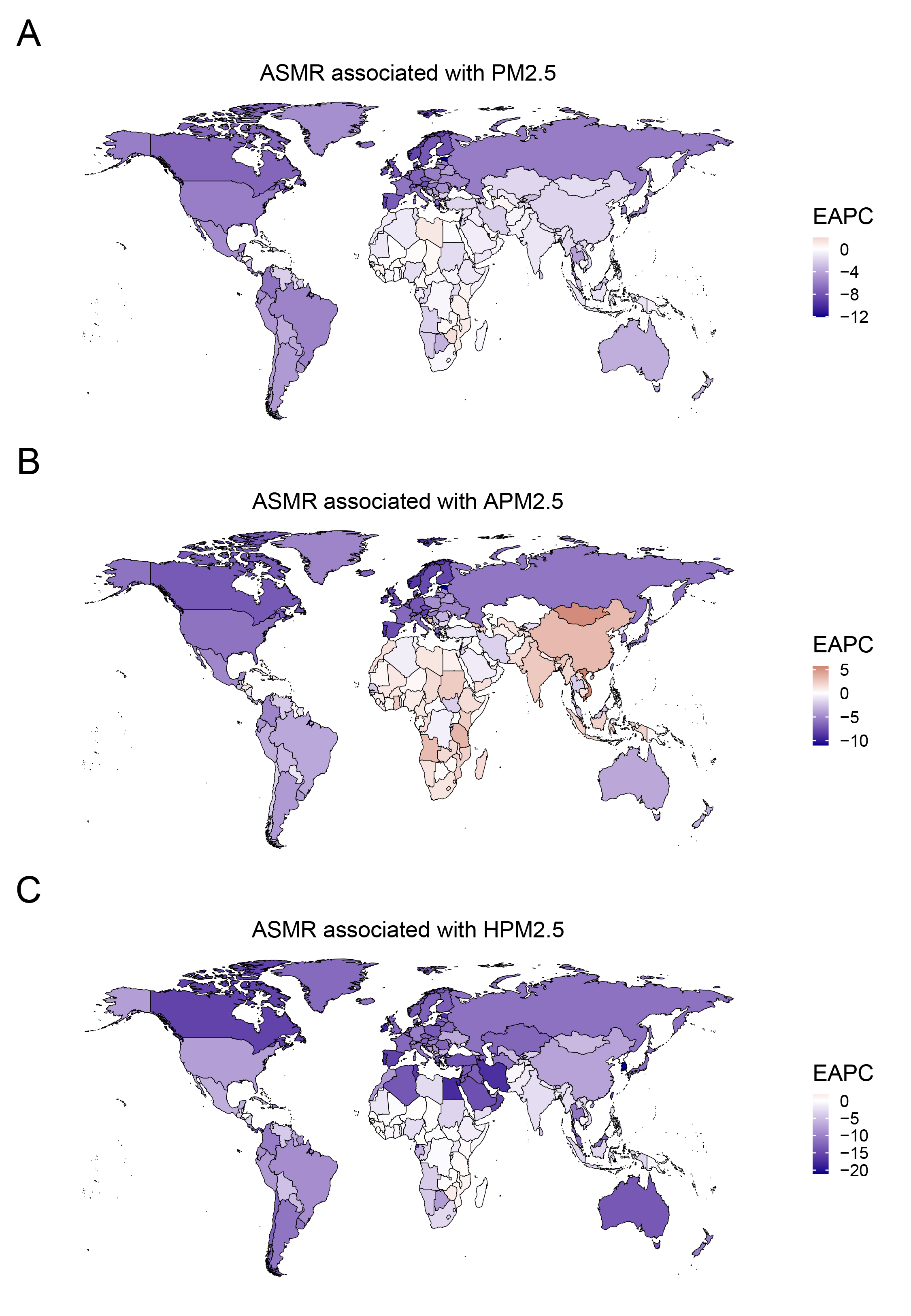

Supplement: SUPPLEMENTARY FIGURE S5 — Temporal trends of ASMR for ischemic stroke attributed to PM2.5 (A), ambient PM2.5 (B), and household PM2.5 (C) air pollution during 1990-2021. ASMR, age-standardized mortality rate; PM, particulate matter. [file Image_5.tif]

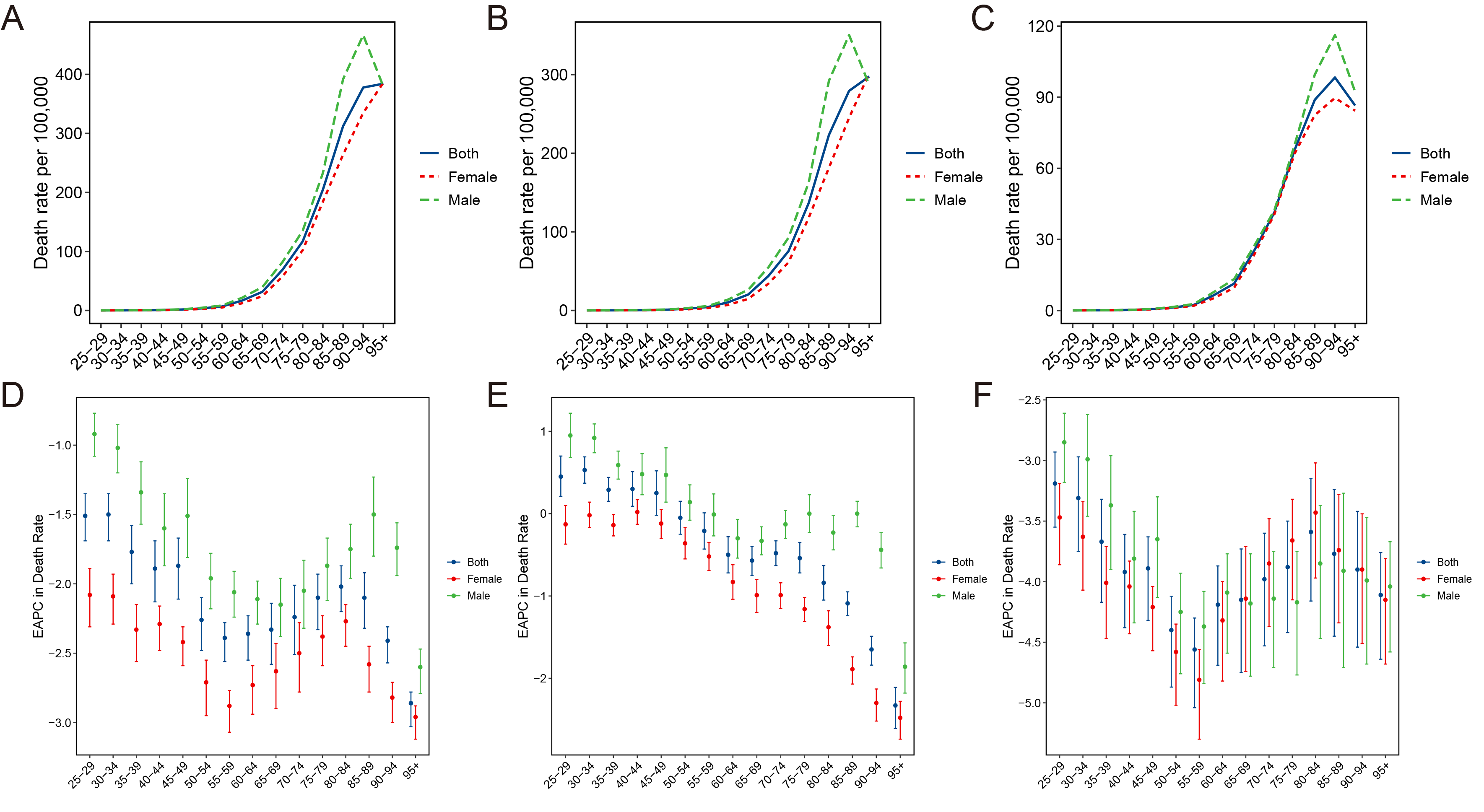

Supplement: SUPPLEMENTARY FIGURE S6 — Age-specific death rate of ischemic stroke attributed to PM2.5 (A), ambient PM2.5 (B), and household PM2.5 (C) air pollution and the corresponding changes in EAPC (D–F), by sex, in 2021. PM, particulate matter; EAPC, estimated annual percentage change. [file Image_6.tif]

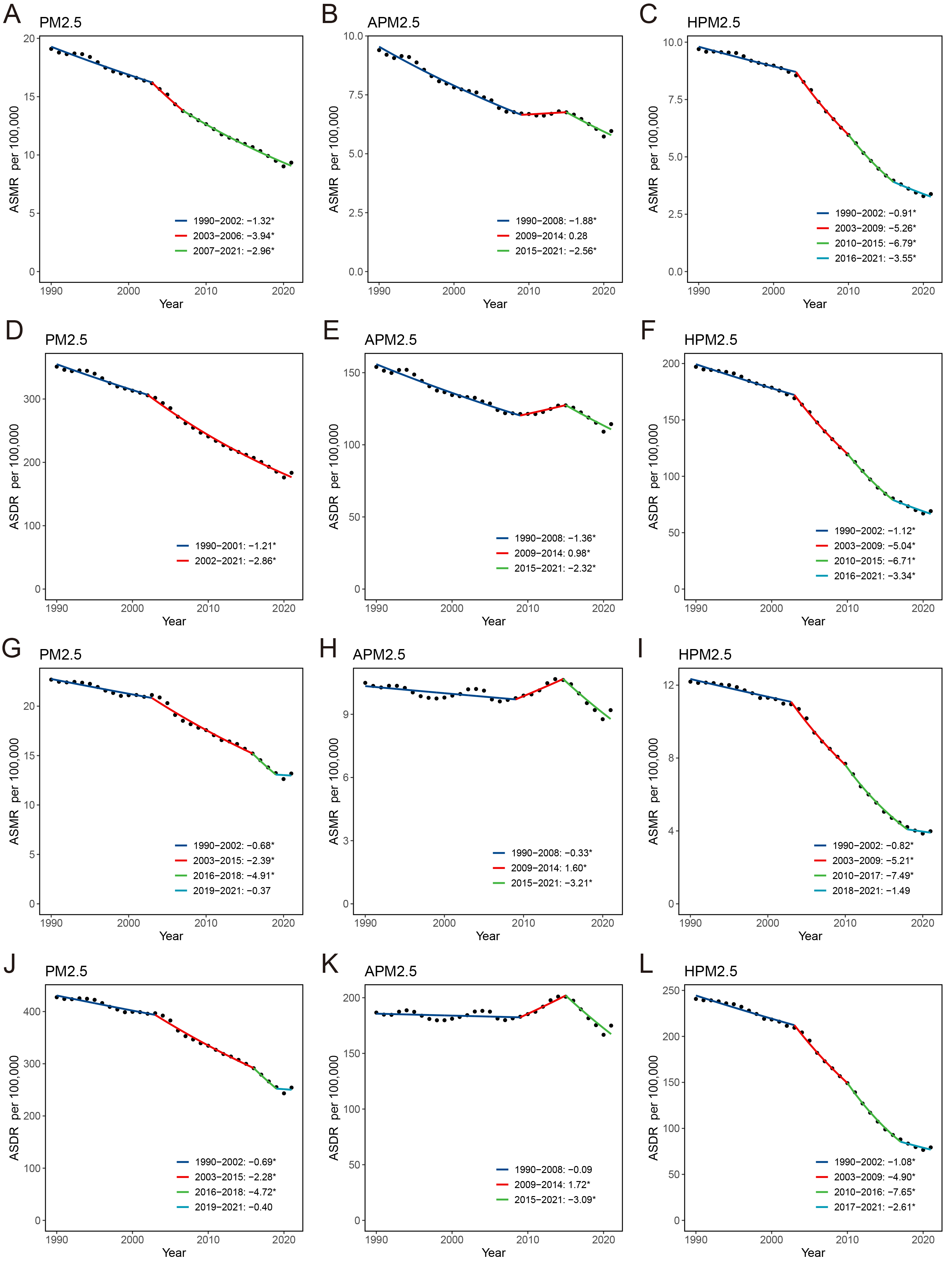

Supplement: SUPPLEMENTARY FIGURE S7 — The global APCs of the ASMR and ASDR for ischemic stroke attributable to PM2.5, ambient PM2.5, and household PM2.5 air pollution, 1990-2021. Female ASMR (A–C) and ASDR (D–F); male ASMR (G–I) and ASDR (J–L) *p<0.05, APC: annual percentage change; ASMR, age-standardized mortality rate; ASDR, age-standardized DALYs rate; PM, particulate matter. [file Image_7.tif]

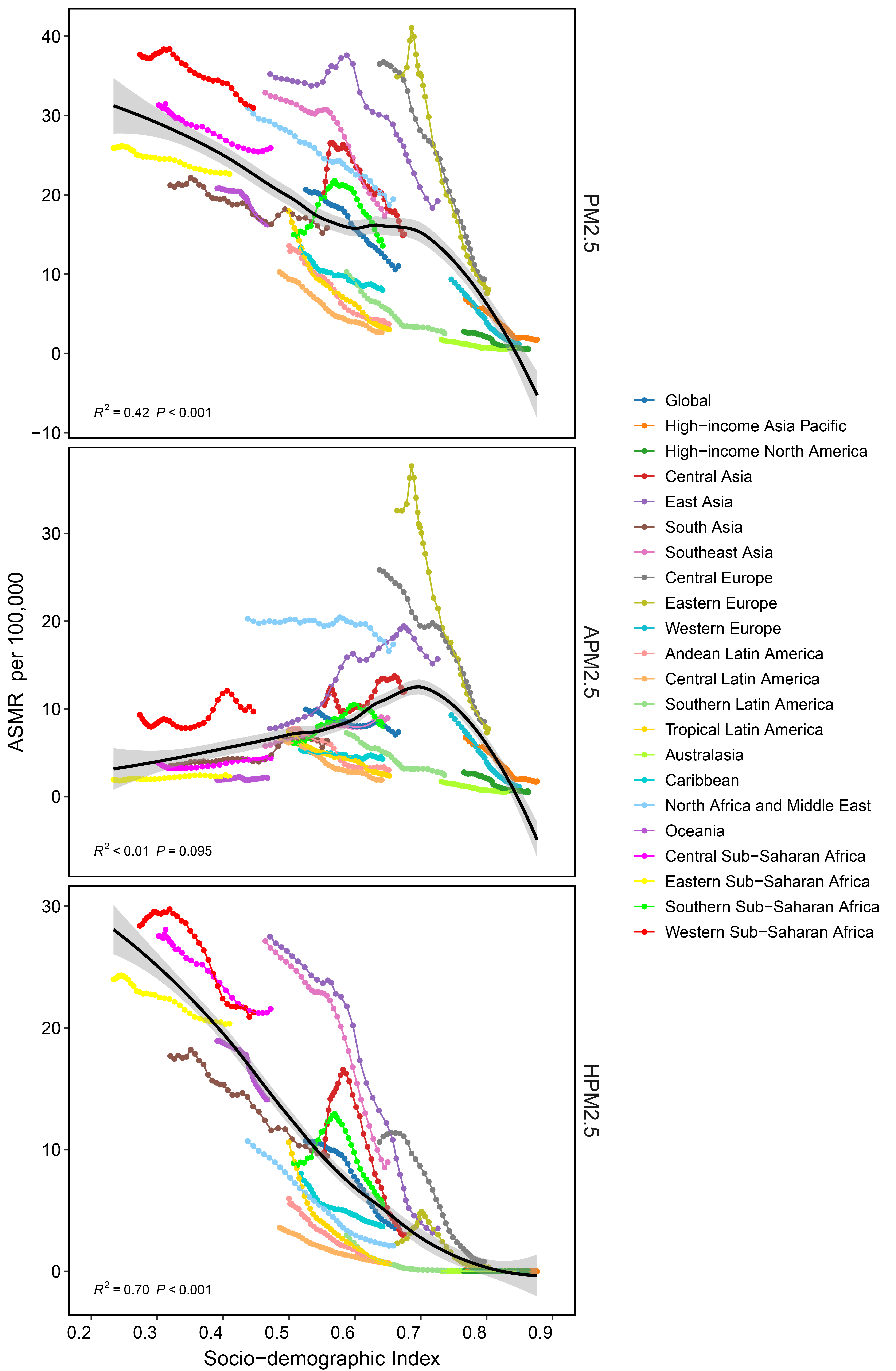

Supplement: SUPPLEMENTARY FIGURE S8 — The correlation between socio-demographic index and ASMR for ischemic stroke attributed to PM2.5, ambient PM2.5, and household PM2.5 air pollution across the GBD regions. ASMR, age-standardized mortality rate; PM, particulate matter; GBD, Global Burden of Disease. [file Image_8.tif]

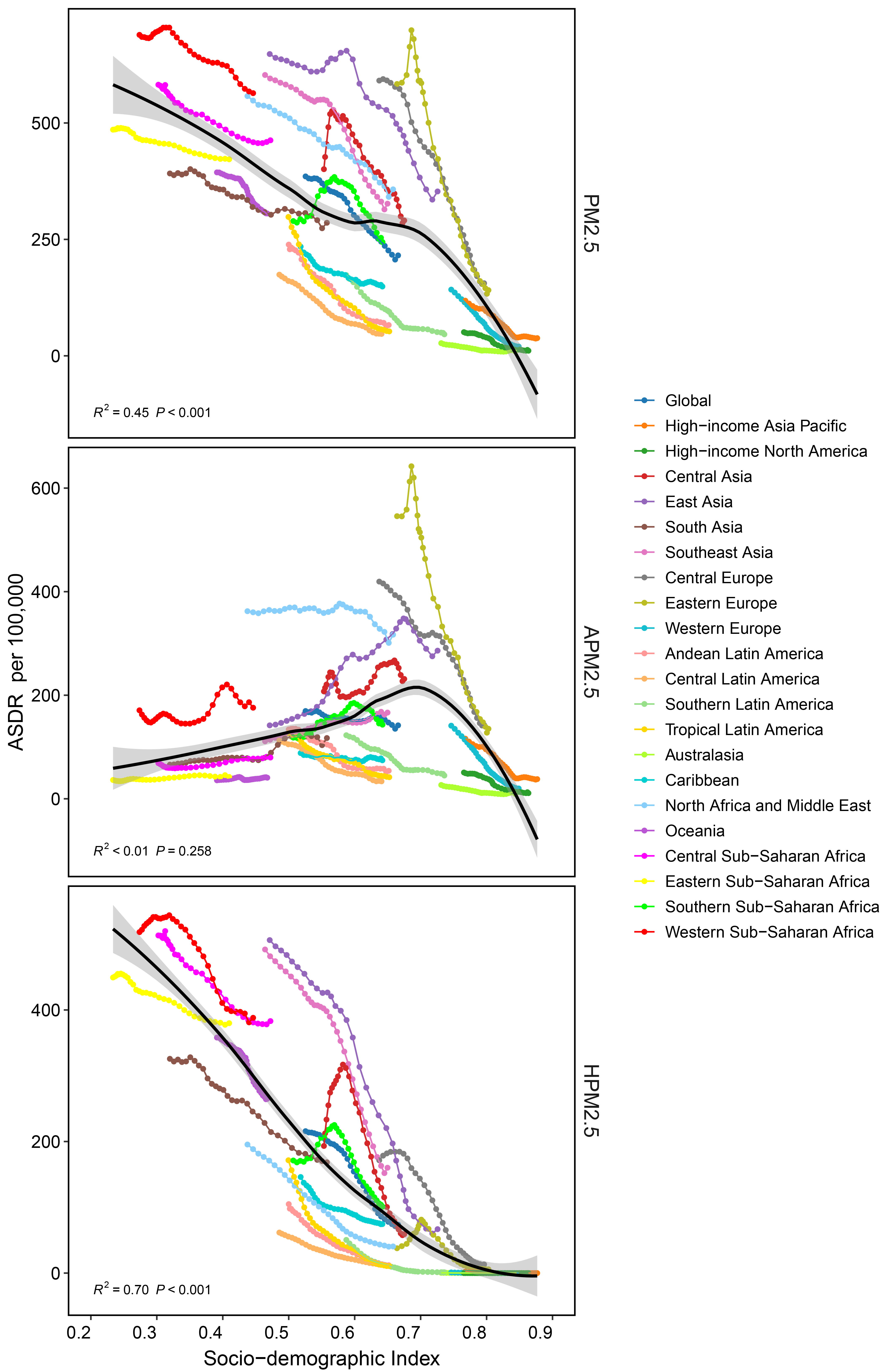

Supplement: SUPPLEMENTARY FIGURE S9 — The correlation between socio-demographic index and ASDR for ischemic stroke attributed to PM2.5, ambient PM2.5, and household PM2.5 air pollution across the GBD regions. ASDR, age-standardized DALYs rate; PM, particulate matter; GBD, Global Burden of Disease. [file Image_9.tif]

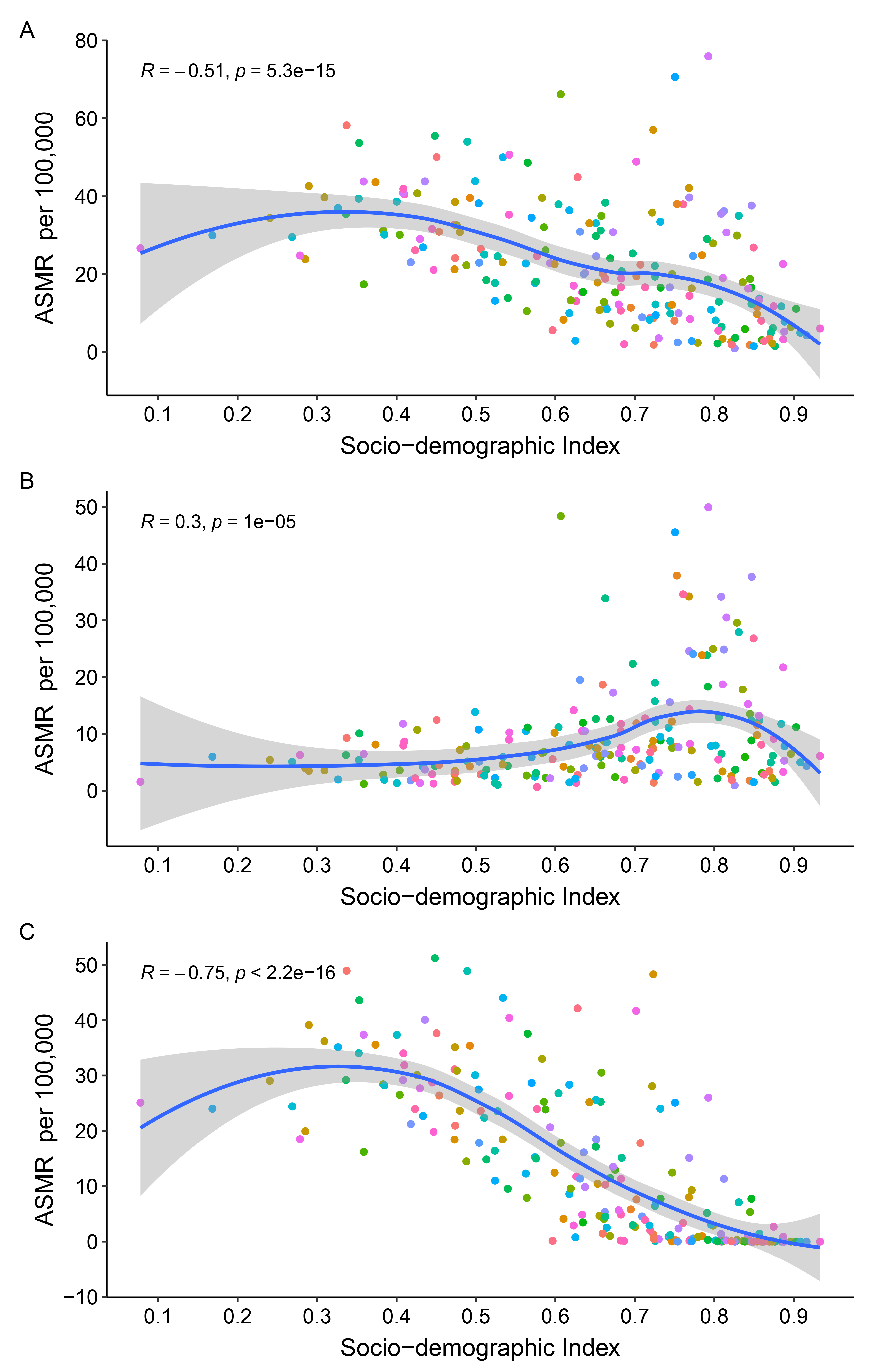

Supplement: SUPPLEMENTARY FIGURE S10 — The relationship between socio-demographic index in 2021 and ASMR in 1990 for ischemic stroke attributed to PM2.5 (A), ambient PM2.5 (B), and household PM2.5 (C) air pollution across 204 countries or territories. ASMR, age-standardized mortality rate; PM, particulate matter. [file Image_10.tif]

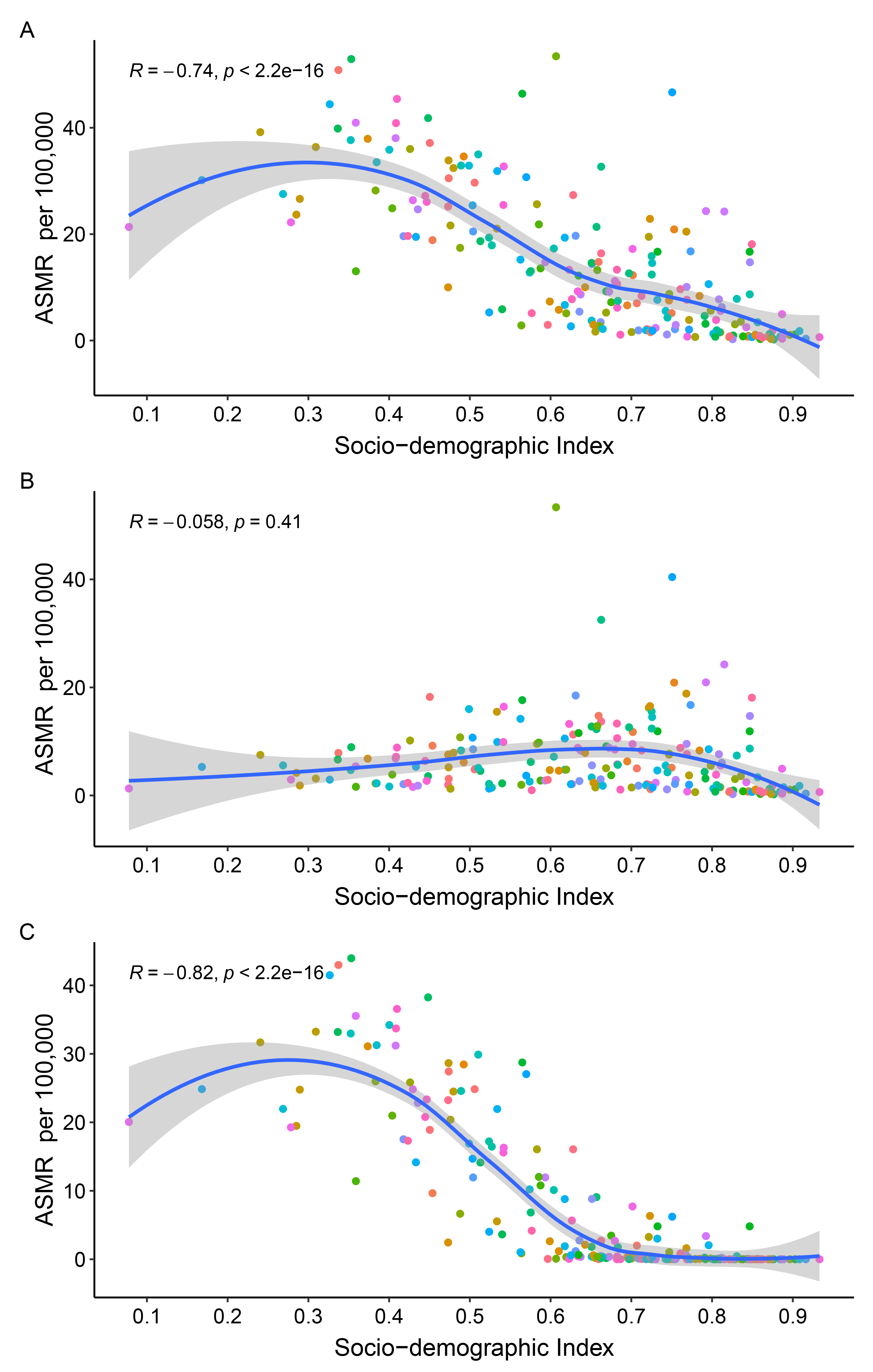

Supplement: SUPPLEMENTARY FIGURE S11 — The relationship between socio-demographic index in 2021 and ASMR in 2021 for ischemic stroke attributed to PM2.5 (A), ambient PM2.5 (B), and household PM2.5 (C) air pollution across 204 countries or territories. ASMR, age-standardized mortality rate; PM, particulate matter. [file Image_11.tif]

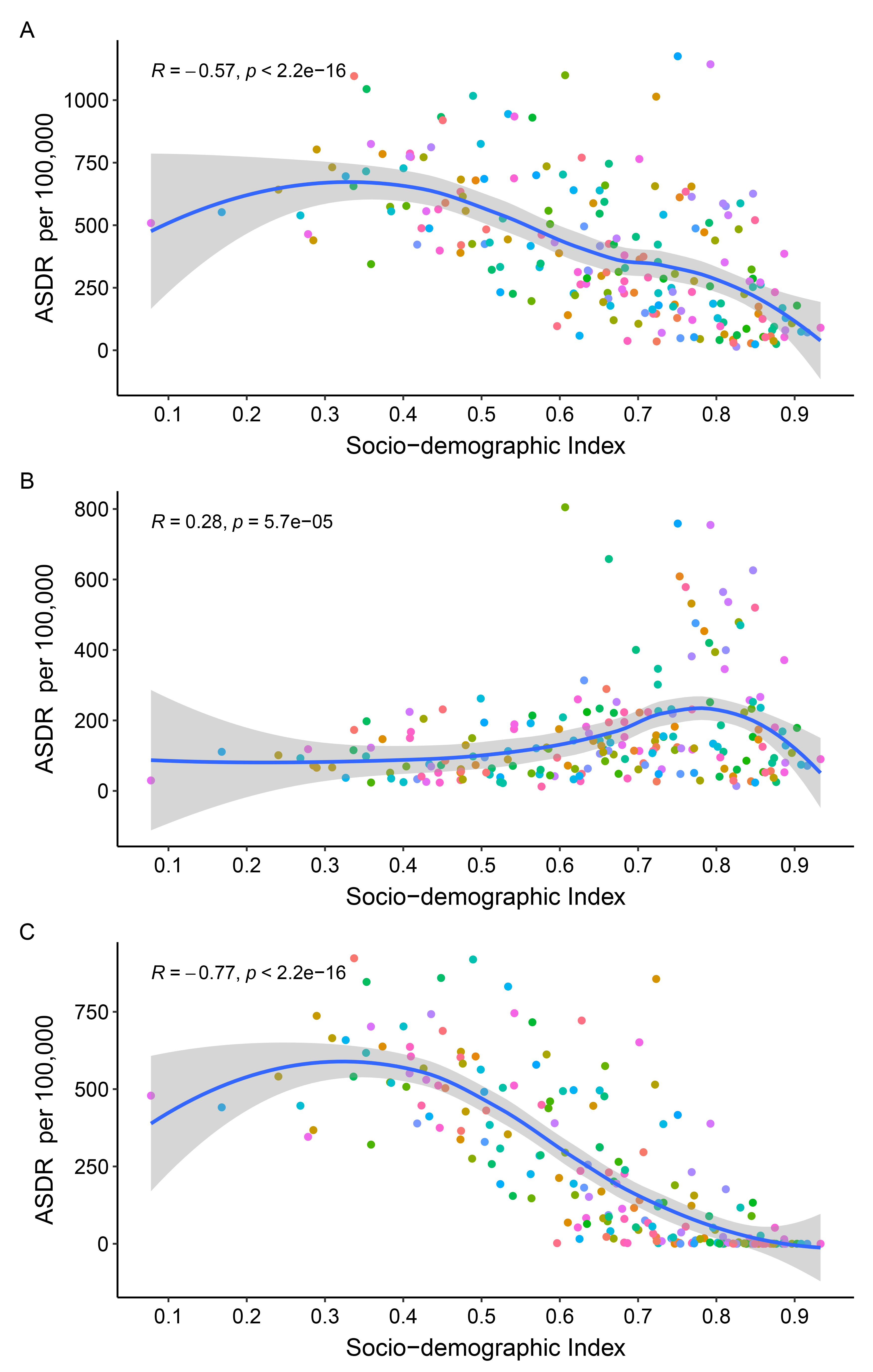

Supplement: SUPPLEMENTARY FIGURE S12 — The relationship between socio-demographic index in 2021 and ASDR in 1990 for ischemic stroke attributed to PM2.5 (A), ambient PM2.5 (B), and household PM2.5 (C) air pollution across 204 countries or territories. ASDR, age-standardized DALYs rate; PM, particulate matter. [file Image_12.tif]

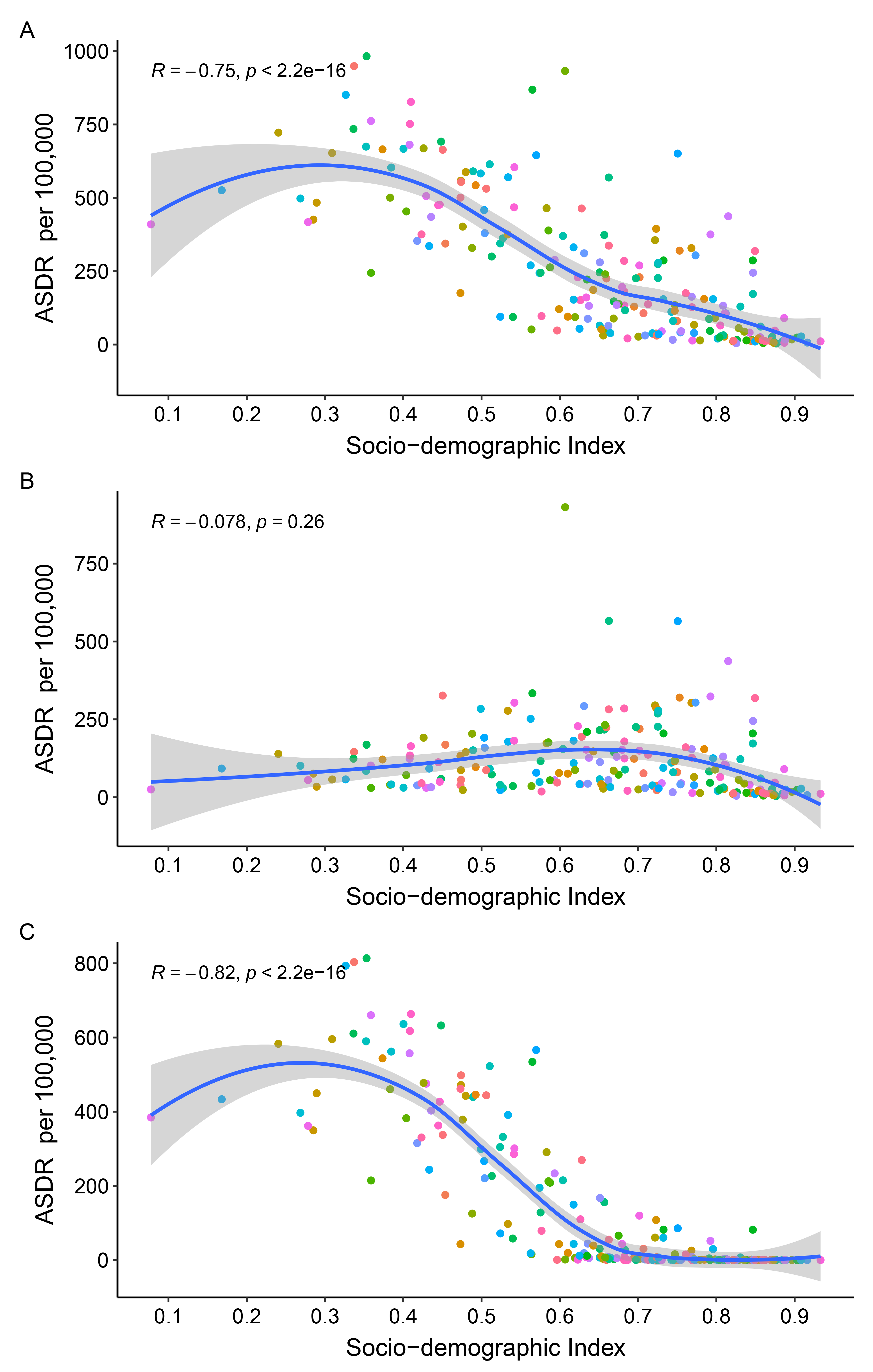

Supplement: SUPPLEMENTARY FIGURE S13 — The relationship between socio-demographic index in 2021 and ASDR in 2021 for ischemic stroke attributed to PM2.5 (A), ambient PM2.5 (B), and household PM2.5 (C) air pollution across 204 countries or territories. ASDR, age-standardized DALYs rate; PM, particulate matter. [file Image_13.tif]
